# Overlapping ETS and CRE Motifs (^G^/_C_CGGAAGTGACGTCA) Preferentially Bound by GABPα and CREB Proteins

**DOI:** 10.1534/g3.112.004002

**Published:** 2012-10-01

**Authors:** Raghunath Chatterjee, Jianfei Zhao, Ximiao He, Andrey Shlyakhtenko, Ishminder Mann, Joshua J. Waterfall, Paul Meltzer, B. K. Sathyanarayana, Peter C. FitzGerald, Charles Vinson

**Affiliations:** * Laboratory of Metabolism, National Cancer Institute, National Institutes of Health, Bethesda, Maryland 20892; † Genetics Branch, National Cancer Institute, National Institutes of Health, Bethesda, Maryland 20892; ‡ Laboratory of Molecular Biology, National Cancer Institute, National Institutes of Health, Bethesda, Maryland 20892; § Genome Analysis Unit, National Cancer Institute, National Institutes of Health, Bethesda, Maryland 20892

**Keywords:** proximal promoters, transcription factor binding sites, co-localization, transcriptional start site, EMSA

## Abstract

Previously, we identified 8-bps long DNA sequences (8-mers) that localize in human proximal promoters and grouped them into known transcription factor binding sites (TFBS). We now examine split 8-mers consisting of two 4-mers separated by 1-bp to 30-bps (X_4_-N_1-30_-X_4_) to identify pairs of TFBS that localize in proximal promoters at a precise distance. These include two overlapping TFBS: the ETS⇔ETS motif (^C^/_G_CCGGAA**G**CGGAA) and the ETS⇔CRE motif (^C^/_G_CGGAA**GTG**ACGTCAC). The nucleotides in bold are part of both TFBS. Molecular modeling shows that the ETS⇔CRE motif can be bound simultaneously by both the ETS and the B-ZIP domains without protein-protein clashes. The electrophoretic mobility shift assay (EMSA) shows that the ETS protein GABPα and the B-ZIP protein CREB preferentially bind to the ETS⇔CRE motif only when the two TFBS overlap precisely. In contrast, the ETS domain of ETV5 and CREB interfere with each other for binding the ETS⇔CRE. The 11-mer (CGGAA**GTG**ACG), the conserved part of the ETS⇔CRE motif, occurs 226 times in the human genome and 83% are in known regulatory regions. *In vivo* GABPα and CREB ChIP-seq peaks identified the ETS⇔CRE as the most enriched motif occurring in promoters of genes involved in mRNA processing, cellular catabolic processes, and stress response, suggesting that a specific class of genes is regulated by this composite motif.

Gene expression is controlled by many genetic and epigenetic elements in a highly coordinated manner, but the DNA sequence of the genome is the ultimate arbiter. Specific DNA sequences in both proximal promoters and more distant regions are bound by sequence-specific DNA binding proteins that regulate gene expression (Smale & Kadonaga 2003; Farnham 2009). Additionally, CpG islands (regions of 300-bps to 3000-bps containing a high frequency of the CG dinucleotide) are frequently located at or near mammalian promoters (Bird 2011). Many experimental (Carninci *et al.* 2006; Johnson *et al.* 2007) and computational methods have been employed to identify biologically relevant transcription factor binding sites (TFBS). The computational methods typically examine DNA sequence enrichment near a biologically defined regulatory region like the transcriptional start site (TSS) (Frith *et al.* 2002; Ohler *et al.* 2002; Kel *et al.* 2003; Bina *et al.* 2004; FitzGerald *et al.* 2004; Marino-Ramirez *et al.* 2004; Matys *et al.* 2006; Pachkov *et al.* 2007; Ji *et al.* 2008; Kharchenko *et al.* 2008; Portales-Casamar *et al.* 2010; Oh *et al.* 2011; Vinson *et al.* 2011). Examination of related mammals has also identified many DNA motifs in promoters that are conserved, suggesting that they may be TFBS, while the 3′UTR have conserved sequences thought to be microRNAs (Xie *et al.* 2005).

In an earlier study, we identified 8-bps long DNA sequences (8-mers) that are localized in human proximal promoters (FitzGerald *et al.* 2004) and Drosophila promoters (FitzGerald *et al.* 2006), and we presented evidence that motifs near the TSS are biologically functional. In human promoters, these sequences were grouped into known TFBS, including SP1, CCAAT, ETS, E-Box, CRE, Box A, NRF1, and TATA. Analyses of promoters with the conservation of DNA sequences among the related mammals greatly enhanced the identification of regulatory motifs (Xie *et al.* 2005).

To identify additional biologically important DNA sequences in human proximal promoters, we analyzed the distribution of discontinuous 8-mers, also called split 8-mers (Vinson *et al.* 2011). Each split 8-mer is composed of two 4-mers separated by 1-bp to 30-bps. If each 4-mer represents a part of a TFBS, this calculation would identify pairs of TFBS that co-occur in the same proximal promoter as observed in other mammalian promoters (FitzGerald *et al.* 2004). Split 8-mer enrichment in promoters declines with increasing distance between the two 4-mers. In contrast, Drosophila contains many split 8-mers in which the 4-mers are separated by 20-bps to 30-bps that localize in promoters (Vinson *et al.* 2011).

This article examines the split 8-mers that localize in human promoters. We extended our previous work with split 8-mers in human promoters (Vinson *et al.* 2011) by evaluating whether the split 8-mers that localize in promoters have a preferred distance between the two 4-mers. This analysis identified an ETS motif overlapping with a CRE motif (ETS⇔CRE) that localizes in proximal promoters. DNA binding experiments show that GABPα and CREB preferentially bind the two TFBS when they overlap and produce the ETS⇔CRE motif enriched in proximal promoters.

## Materials and Methods

### Dataset generation

From University of California Santa Cruz Genome Bioinformatics website (http://genome.ucsc.edu/), we obtained the DNA sequence data for RefSeq genes in the Golden Path Human Genome Assembly with annotated TSS, representing sequences from –1,000 bp to +500 bp relative to the TSS. The initial dataset contained 26,431 promoters. The set was further processed to improved relevance and the validity of the analysis using the following criteria. First, for promoters with 100% identical sequences, only one copy of them was kept (5483 promoters were removed). Second, promoters containing unknown nucleotides (N) of at least 150 bps were removed (8 promoters). Third, promoters with duplicated RefSeq numbers were removed (411 promoters). Fourth, of the remaining 20,529 promoters, 18,451 were determined to have unique sequences, whereas 2078 promoters had duplicated sequences shared among themselves. Among these 2078 promoter sequences, 68 had more than 10 overlapping duplicated regions of at least 250 bps with other promoter sequences and were deleted from the analysis. One thousand five hundred thirty-five (1535) promoter sequences contained closely identical sequences among themselves, and they comprised 701 unique groups (pairs in most cases); only 701 “representative” promoters were kept for the analysis. An additional 475 promoters were kept for the analysis, although they did have some mixed overlapping sequencing. This allowed us to retain only 1176 out of these 2078 promoters. Fifth, two thousand four hundred eighty-four (2484) promoters had start of the coding sequences (translational start sites) within 30-bps of the TSS, and these promoters were excluded from the following analysis. Finally, a set of 17,143 promoters (18,451 + 1,176 − 2,484) was obtained and considered for the analysis.

### Analysis of split 8-mers distributions

There are 4^8^ discontinuous non-degenerative 8-mers (X_4_-N*_k_*-X_4_; N denotes any arbitrary nucleotides and *k* denotes spacing between two 4-mers), and of these, *ξ*4^4^ are palindromes and $\left(4^{8}-\xi 4^{4}\right)$ are non-palindromes, where each sequence and its complement is represented and *ξ* = 1 if *k* is even and 0 if odd. Thus, the number of 8-mers can be reduced to$\left(4^{8}-\xi 4^{4}\right)/2+\xi 4^{4}=4^{4}\frac{\left(4^{4}+\xi \right)}{2}$. Those 32,896 or 32,768 8-mers were automatically generated by a custom-made program. The promoter set was searched against them, and final distributions were generated. To analyze the data, we divided 1500-bps into 75 bins each containing 20-bps, numbering bin 1 [–1000 bp; –981 bp] to bin 75 [+481 bp; +500 bp]. We determined the number of times the first nucleotide of a studied DNA sequence (or the last of its complement) occurred within each 20-bps bin. To detect and quantify non-uniform distributions (localization) and the probability of non-uniformity of split 8-mers, we determined localization factor (LF) and *P*-value as described previously (FitzGerald *et al.* 2004; Vinson *et al.* 2011).

### Molecular modeling

The molecular model of the ETS and CREB dimer interacting with a single chain DNA with a specific base pair sequence of CCGGAAGTGACGTCA was built by using two PDB structures, the ETS-1 protein bound to an ETS site (PDB ID: 1K79) (Garvie *et al.* 2001) and the CREB dimer bound to the CRE (PDB ID: 1DH3) (Schumacher *et al.* 2000). The 10 nucleotides (shown underlined) of the E chain of the DNA (TAGTGCCGGAA**ATG**T) of 1K79 were aligned to the 10 nucleotides (shown underlined) in the B chain of the DNA (CCTTGG**CTG**ACGTCAGCCAAG) of 1DH3, using Chimera visualization software (Pettersen *et al.* 2004). This alignment also results in the nucleotides ATG (shown in bold) of 1K79 aligning with the nucleotides CTG (shown in bold) of 1DH3. The ETS-1 protein and the complementary strand (F chain) of DNA of 1K79 were carried along with the E chain of its DNA during this alignment. From this aligned structures, the first 10 nucleotides (CCTTGGCTGA) and their base pairs in the complimentary chain in the 1DH3 structure were deleted. The remaining chains containing the nucleotides TAGTGCCGGAAATGT of 1K79 and the nucleotides CGTCAGCCAAG of 1DH3 were covalently linked to one another using Chimera software to form one long chain of DNA with the sequence TAGTGCCGGAA**A**TG**T**CGTCAGCCAAG. Similarly, its complimentary DNA chain was also built. The 12^th^ and 15^th^ bases in this long chain (shown in bold) were mutated to G and A bases, respectively, and the final complex containing this long DNA and the ETS and CRE was subjected to an energy minimization using the Discovery Studio (Accelrys Software) molecular modeling software.

### Electrophoretic mobility shift assay (EMSA)

EMSA was performed similarly as described previously (Rishi *et al.* 2010). GABPα and CREB proteins were *in vitro* translated using PURExpress *In Vitro* Protein Synthesis Kit (New England Biolabs, USA) according to manufacturer instructions. The T7 expression plasmids containing the DNA binding domain of GABPα (Badis *et al.* 2009) or the B-ZIP domain of CREB (Ahn *et al.* 1998) was used as the template DNA. GABPα has a GST-tag at the N-terminus. The protein concentrations were estimated by Western blot using purified GST-CREB or CREB with known concentrations as concentration standards. *In vitro* translated proteins were mixed with 7 pM ^32^P end-labeled double-stranded oligonucleotides containing variants of ETS and CREB binding sites in the gel shift buffer (0.5 mg/ml BSA, 10% glycerol, 2.5 mM DTT, 12.5 mM K_2_HPO_4_-KH_2_PO_4_, pH 7.4, 0.25 mM EDTA). The final volume of the reaction was adjusted to 20 µl. For regular EMSA, the reactions were incubated at 37° for 20 min, followed by cooling at room temperature for 5 min before loading. For supershift experiments, the reactions were first incubated at 37° for 20 min without antibodies. Antibodies (catalog # sc-186, sc-459, or sc-2027, Santa Cruz Biotechnology, USA) were then added, and the reactions were incubated on ice for 30 min, followed by incubation at room temperature for 15 min before loading. 10 µl samples were resolved on 7.5% PAGE at 150 V for 1.5 hr in the 1x TBE buffer (25 mM Tris-boric acid, 0.5 mM EDTA). Sequences of oligonucleotides used for EMSA experiments are listed in Table 1. For EMSA using ETV5 and CREB, we used purified proteins containing the DNA binding domain of ETV5 or the B-ZIP domain of CREB.

**Table 1:** DNA probe sequences for EMSA (binding sites underlined)

Probe	Sequence (5′ to 3′)
ETS⇔CRE	GTCAGTCAGACCGGAAGTGACGTCATATCGGTCAG
ETS-1−CRE	GTCAGTCAGACCGGAATGACGTCATATCGGTCAGT
ETS+1−CRE	TCAGTCAGACCGGAAGTTGACGTCATATCGGTCAG
ETS+2−CRE	TCAGTCAGACCGGAAGTGTGACGTCATATCGGTCA
ETS+3−CRE	CAGTCAGACCGGAAGTGGTGACGTCATATCGGTCA
ETSm−CRE	GTCAGTCAGAGGCCAAGTGACGTCATATCGGTCAG
ETS−CREm	GTCAGTCAGACCGGAAGTGTGCACATATCGGTCAG
ETSm−CREm	GTCAGTCAGAGGCCAAGTGTGCACATATCGGTCAG

### Motif enrichment using ChIP-seq peaks

For motif analysis, we used published 6442 GABPα ChIP-seq peaks from human Jurket cell line (Valouev *et al.* 2008) and 3998 CREB ChIP-seq peaks from mouse in GC1 cells (Martianov *et al.* 2010). For motif detection, we used MEME (Machanick & Bailey 2011) and the peak-motifs package of the Regulatory Sequence Analysis Tools (RSAT) (Thomas-Chollier *et al.* 2011). Two thousand eight hundred thirty-four (2834) CREB binding promoters, which were obtained from the ChIP-chip data on human HEK293T cells in three time points (Zhang *et al.* 2005), were mapped to human (hg18), which successfully resulted in 2384 promoters bound by CREB. For *de novo* motif prediction, we used 1463 common binding regions of human CREB ChIP-chip and GABPα ChIP-seq data.

### PhyloP conservation

Base by base PhyloP score or the *P*-values for conservation or acceleration *P*-values based on an alignment and a model of neutral evolution among the 36 mammalian genomes were (Pollard *et al.* 2010) downloaded from UCSC database (http://genome.ucsc.edu/). PhyloP scores for each nucleotide in the motif, including 15-bps upstream and 15-bps downstream of each occurrence in the genome, were averaged for all occurrences of each motif.

### Gene Ontology analysis

Gene Ontology (GO) analysis was performed using DAVID (http://david.abcc.ncifcrf.gov/). Go terms with *P*-values < 0.01 were considered as significantly enriched GO terms. Additionally, Benjamini-Hochberg corrected *P*-values < 0.01 were considered for the analysis with *in vivo* ChIP data.

## Results

### Split 8-mers that localize in human proximal promoters

We aligned human promoters relative to the TSS and determined the distribution of split 8-mers in the promoter region. The split 8-mers consist of two 4-mers separated by 1-bp to 30-bps (X_4_-N_1-30_-X_4_). We considered the promoter region from −1000-bps to +500-bps relative to the TSS and divided the 1500-bp region into 75 bins of 20-bps each. We used a human DNA promoter sequence set obtained from UCSC and removed promoters containing repetitive sequences, resulting in a set of 17,143 promoter sequences (see *Materials and Methods*). The distribution of each split 8-mer in promoters was determined and a measure of non-uniform distribution termed “localization factor” (LF) was calculated (Vinson *et al.* 2011). The statistical significance of the non-random distribution of LF was determined by calculating a probability value (*P*-value) for each split 8-mer.

**Figure 1:**
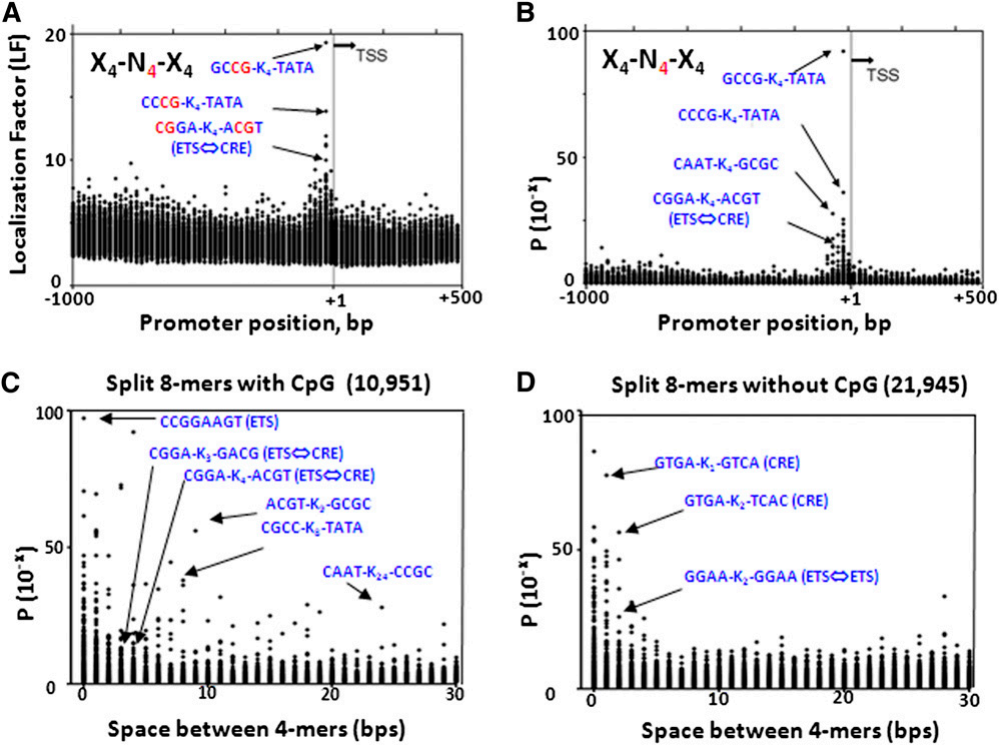
(A and B) LF and probability for split 8-mers with a 4-bp insert (X_4_-N_4_-X_4_). (C and D) For each 8-mer (X_4_-N_0-30_-X_4_), we determine which insert length produced the largest LF and plot that value in the column representing that insert length. (C) LF for the 12,547 continuous 8-mers and 10,951 split 8-mers containing the CG dinucleotide. We plot that –log *P*-value at the insert length with the highest LF. (D) Same as (C) but for all non-CG containing 8-mers, the 20,349 continuous 8-mers, and 21,945 split 8-mers with insert length from 1-bp to 30-bps.

Many continuous 8-mers (X_4_-N_0_-X_4_) are enriched in proximal promoters (−120-bps to the TSS) (supporting information, Figure S1, A and B, and Table S1) (FitzGerald *et al.* 2004, 2006; Xie *et al.* 2005; Vinson *et al.* 2011). In contrast, fewer split 8-mers with an insert length of 4-bps (X_4_-N_4_-X_4_) localize in proximal promoters (FitzGerald *et al.* 2004; Vinson *et al.* 2011) (Figure 1, A and B). As insert length increases, preferential localization of split 8-mers in the proximal promoter decreases for both CG- and non-CG split 8-mers and is much more pronounced for the non-CG 8-mers (Figure 1, C and D).

The most localizing split 8-mer sequences with an insert length of 1-bps and 2-bps both represent the CRE motif (Figure 1D and Table S1), suggesting that the CRE is 10-bps long (GTGACGTCAC). The most localizing sequence with both a 3-bps and 4-bps insert are a CG-rich 4-mer followed by TATA (CCGG-N_3_-TATA and GCCG-N_4_-TATA), sequences previously identified that function in proximal promoters (Lagrange *et al.* 1998). These split 4-mers are not strand specific, indicating that the CG-rich 4-mer can be either before or after the strand-specific TATAA (FitzGerald *et al.* 2004). Virtually all the localizing split 8-mers with an insert length of 5-bps or more contain the CG dinucleotide (Figure 1, C and D). The 20 most localizing split 8-mers with insert length of 0-bps, 2-bps, 4-bps, and 5-bps to 30-bps are presented in Table S1.

### Split 8-mers that localize in promoters at a unique insert length

The split 8-mers that localize in proximal promoters were grouped into three classes (Table S1): (i) split 8-mers with a short insert length of 1-bps or 2-bps representing a single TFBS (Figure S2, A–D); (ii) split 8-mers that localize in proximal promoters at many insert lengths representing co-localizing TFBS, each represented by a single 4-mer (Figure S2, E–H); and (iii) split 8-mers that localize in proximal promoters at a specific insert length. These include CGGA-N_4_-ACGT, which represents an ETS motif and a CRE motif, and unidentified sequences; *e.g.* GGGA-N_2_-TGTA (Figure S2, I and J).

**Figure 2:**
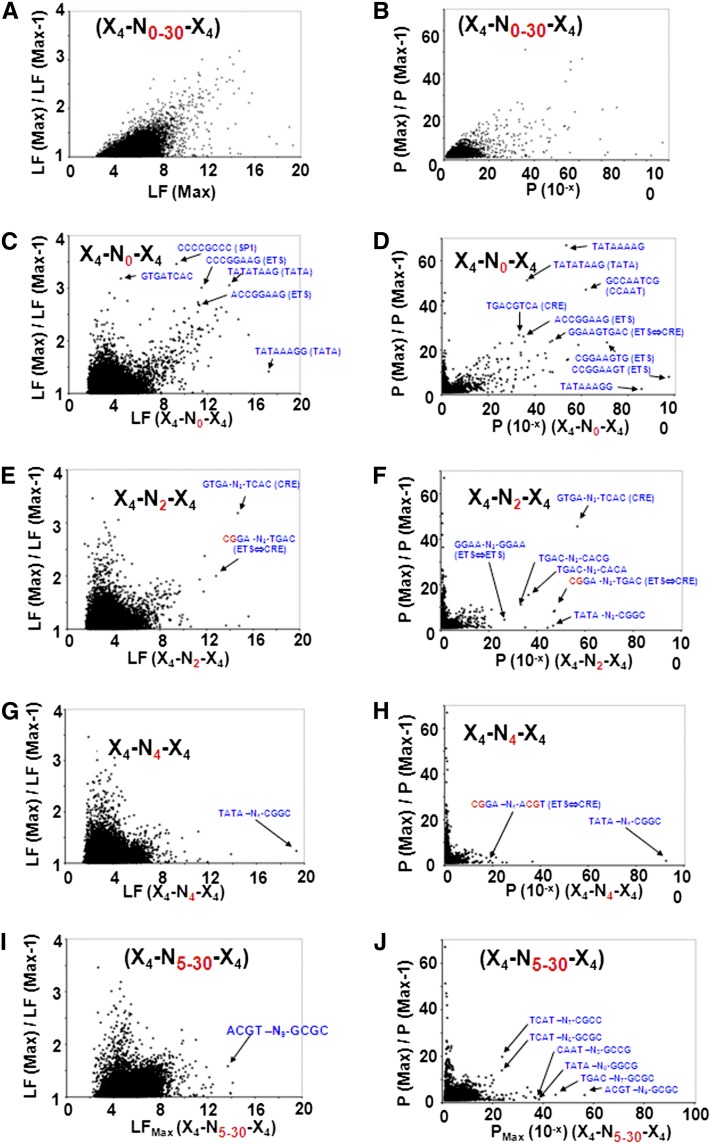
Identification of split 8-mers that localize in promoters only at a unique insert length. (A) The maximum LF for all 8-mers (X_4_-N_0-30_-X_4_) is plotted on the horizontal axis *vs.* maximum LF for 8-mers with an insert length from 0-bps to 30-bps [LF(Max)] divided by the second highest LF [LF(Max-1)]. The points at the top right of the plot represent 8-mers that localize in promoters only at one inset length. (B) Probability (P) (*P* = 10^–x^) of the LF being non-random. To identify the insert length that produces unique localization in promoters, the horizontal axis shows the probability of LF for split 8-mers for specific insert lengths. (C and D) Localization of continuous 8-mers (X_4_-N_0_-X_4_) in proximal promoters only when the inset length is 0-bps. (E and F) Localization of split 8-mers with insert length of 2-bps (X_4_-N_2_-X_4_) in proximal promoters only when the inset length is 2-bps. (G and H) Localization of split 8-mers with insert length of 4-bps (X_4_-N_4_-X_4_) in proximal promoters only when the inset length is 4-bps. (I and J) Unique localization of split 8-mers with insert length ranging from 5-bps to 30-bps (X_4_-N_5-30_-X_4_).

To identify split 8-mers that localize in proximal promoters at only a precise insert length, the max LF for all split 8-mers with insert lengths from 0-bps to 30-bps (X_4_-N_1-30_-X_4_) were determined and compared with the ratio of max LF to the second highest LF (Figure 2, A and B). A close to 1 ratio of max LF to the second highest LF indicates localization of split 8-mers at various insert lengths, whereas a ratio with higher values is indicative of split 8-mers that are localized at a precise insert length. Both kinds of sequences are observed for 8-mers with a high LF. To identify the insert length that produces the precisely positioned pairs of 4-mers, we examined each insert length. Continuous 8-mers (X_4_-N_0_-X_4_) have many sequences with a high LF and large ratio (LF(MAX)/LF(MAX-1). These sequences are the TFBS previously described that localize in proximal promoters (FitzGerald *et al.* 2004). The two 4-mers (TGAC and GTCA) that create the CRE (TGACGTCA) motif preferentially localize in promoters when the insert length is 0-bps (Figure 2D and Figure S2, A and B). Similar results were obtained for the ETS motif (Figure S2, C and D). When we examined split 8-mers with an insert length of 2-bps, fewer 8-mers had both a high LF and ratio (Figure 2, E and F). These include GTGA-N_2_-TCAC, representing the CRE; CGGA-N_2_-TGAC, representing overlapping ETS and CRE TFBS (ETS⇔CRE) (CGGA*AG*TGAC); and GGAA-N_2_-GGAA, representing an ETS motif overlapping with a second ETS motif (ETS⇔ETS) (GGAA*GC*GGAA) (Table S1 and Figure 2). A systematic analysis of the human promoters using comparative genomics for the detection of regulatory motifs also identified an unannotated motif GGAANCGGAANY (Xie *et al.* 2005), which is essentially the ETS⇔ETS motif. Insert length of 4-bps produced even fewer sequences that are precisely localized (Figure 2, G and H). Insert length of 5-bps to 30-bps identified many 8-mers with a high LF but a low ratio, indicating that they are co-occurring in promoters at many insert lengths (Figure 2, I and J).

**Figure 3:**
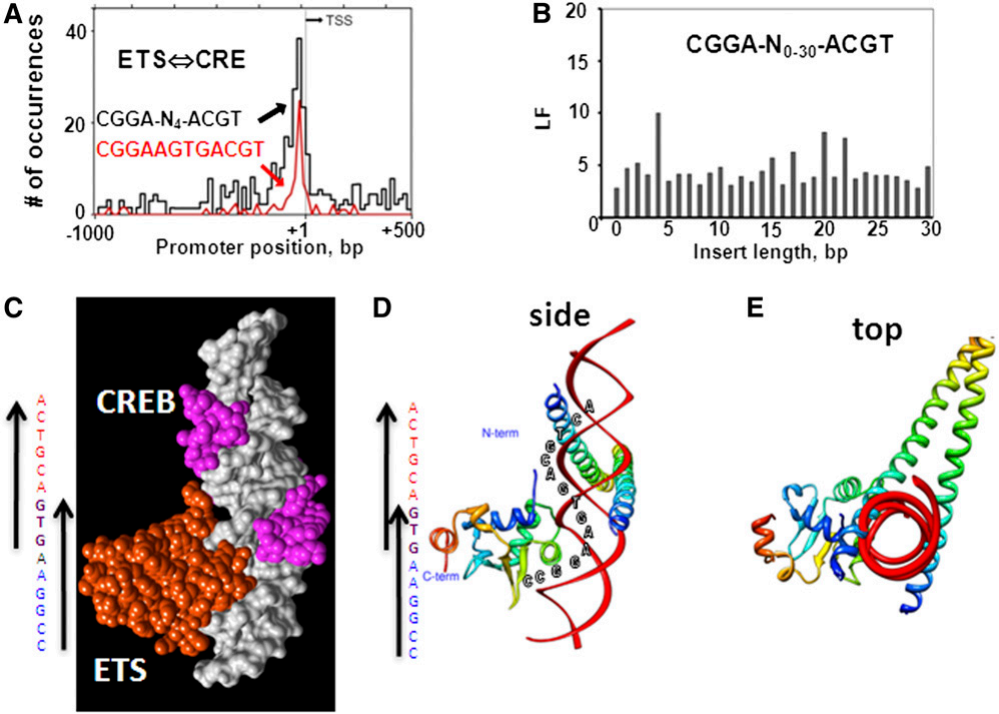
The ETS⇔CRE motif. (A) Distribution of the split 8-mer CGGA-N_4_-ACGT and the 12-mer CGGAA**GTG**ACGT in human promoters. (B) LF for CGGA-N_4_-ACGT from insert size of 0-bps to 30-bps. (C) Space-filling model of ETS and CREB proteins binding to ETS⇔CRE (GCGGAA**GTG**ACGTCA). Note the 3-bp overlap of the two TFBS. (D and E) Ribbon presentation of ETS and CREB proteins binding to ETS⇔CRE motif from the side and top relative to DNA.

This analysis identified many split 8-mers with distinctive distributions; we focused our analysis on the overlapping ETS and CRE motifs. The distribution of the ETS⇔CRE motif split 8-mer CGGA-N_4_-ACGT shows localization in proximal promoters (Figure 3A). The split 8-mer CGGA-N_0-30_-ACGT preferentially localizes in proximal promoters when separated by 4-bps, with the continuous 12-mer CGGAA**GTG**ACGT being the most localizing and abundant (Figure 3, A and B). More modest localization is observed at 20-bps and 22-bps, which has not been evaluated. This sequence contains both the ETS motif (CGGAA**GTG)** and the CRE motif (**GTG**ACGT). The **GTG** trinucleotide is common to both the ETS and CRE motifs. These TFBS overlap to produce the ETS⇔CRE motif. The full ETS⇔CRE motif would be the two 16-mers ^C^/_G_CGGAA**GTG**ACGTCAC that occur five times in the human genome (Table 2). There are more than 4×10^9^ 16-mers, and thus, each 16-mer would be expected to occur by chance only about once in a vertebrate genome of ∼3×10^9^ bps.

**Table 2:** Occurrence of specific motifs in human genome, promoters, CpG islands, and housekeeping DHS regions

			Whole Genome	Promoter	Proximal Promoter	CpG Islands	Housekeeping DHS	All DHS	Tissue-specific DHS
Motifs	N-mers	DNA Sequence	# Unmasked (100%)	(−1000…500) (0.8%)	(−200…60) (0.1%)	(0.7%)	(0.2%)	(8.9%)	(8.7%)
CRE	8-mer	TGACGTCA	10,355	713 (7%)	431 (4%)	757 (7%)	458 (4%)	3,110 (30%)	2,652 (26%)
CRE	9-mer	GTGACGTCA	4,301	654 (15%)	427 (10%)	772 (18%)	449 (10%)	1,890 (44%)	1,441 (34%)
CRE	10-mer	GTGACGTCAC	644	167 (26%)	116 (18%)	217 (34%)	117 (18%)	356 (55%)	239 (37%)
ETS	8-mer	CCGGAAGT	13,975	1,654 (12%)	1,030 (7%)	1,611 (12%)	1,136 (8%)	4,384 (31%)	3,248 (23%)
ETS	8-mer	CGGAAGTG	16,846	1,631 (10%)	980 (6%)	1,761 (10%)	1,073 (6%)	5,068 (30%)	3,997 (24%)
ETS	9-mer	CCGGAAGTG	4,913	868 (18%)	568 (12%)	852 (17%)	587 (12%)	1,887 (38%)	1,300 (26%)
ETS	9-mer	GCGGAAGTG	4,010	469 (12%)	282 (7%)	563 (14%)	316 (8%)	1,370 (34%)	1,054 (26%)
ETS	9-mer	CGGAAGTGA	4,675	465 (10%)	298 (6%)	446 (10%)	343 (7%)	1,456 (31%)	1,113 (24%)
ETS	10-mer	CGGAAGTGAC	1,030	227 (22%)	162 (16%)	227 (22%)	180 (17%)	458 (44%)	278 (27%)
ETS⇔CRE	11-mer	CGGAAGTGACG	226	157 (69%)	124 (55%)	164 (73%)	134 (59%)	186 (82%)	52 (23%)
ETS⇔?	11-mer	CGGAAGTGACA	335	13 (4%)	9 (3%)	12 (4%)	9 (3%)	88 (26%)	79 (24%)
ETS⇔?	11-mer	CGGAAGTGACC	197	21 (11%)	7 (4%)	23 (12%)	18 (9%)	71 (36%)	53 (27%)
ETS⇔AP1	11-mer	CGGAAGTGACT	267	36 (13%)	12 (4%)	28 (10%)	19 (7%)	111 (42%)	92 (34%)
ETS⇔AP1	11-mer	CGGAAGTGAGT	250	20 (8%)	11 (4%)	19 (8%)	12 (5%)	91 (36%)	79 (32%)
ETS⇔CRE	12-mer	CGGAAGTGACGT	93	70 (75%)	53 (57%)	71 (76%)	60 (65%)	84 (90%)	24 (26%)
ETS⇔CRE	12-mer	CGGAAGTGACGC	81	62 (77%)	53 (65%)	67 (83%)	53 (65%)	68 (84%)	15 (19%)
ETS⇔CRE	13-mer	GGAAGTGACGTC	33	23 (70%)	17 (52%)	25 (76%)	19 (58%)	29 (88%)	10 (30%)
ETS⇔CRE	13-mer	CCGGAAGTGACGT	35	26 (74%)	17 (49%)	27 (77%)	22 (63%)	34 (97%)	12 (34%)
ETS⇔CRE	13-mer	GCGGAAGTGACGT	32	28 (88%)	25 (78%)	25 (78%)	24 (75%)	29 (91%)	5 (16%)
ETS⇔CRE	13-mer	CCGGAAGTGACGC	52	42 (81%)	36 (69%)	44 (85%)	32 (62%)	46 (88%)	14 (27%)
ETS⇔CRE	13-mer	GCGGAAGTGACGC	19	15 (79%)	12 (63%)	14 (74%)	14 (74%)	15 (79%)	1 (5%)
ETS⇔AP1	13-mer	CGGAAGTGACTCA	17	3 (18%)	0 (0%)	0 (0%)	0 (0%)	13 (76%)	13 (76%)
ETS⇔AP1	13-mer	CGGAAGTGAGTCA	22	0 (0%)	0 (0%)	0 (0%)	0 (0%)	16 (73%)	16 (73%)
ETS⇔CRE	14-mer	CGGAAGTGACGTCA	18	13 (72%)	11 (61%)	15 (83%)	12 (67%)	18 (100%)	6 (33%)
ETS⇔CRE	15-mer	CGGAAGTGACGTCAC	7	5 (71%)	4 (57%)	6 (86%)	4 (57%)	7 (100%)	3 (43%)
ETS⇔CRE	15-mer	CCGGAAGTGACGTCA	7	4 (57%)	2 (29%)	4 (57%)	3 (43%)	7 (100%)	4 (57%)
ETS⇔CRE	15-mer	GCGGAAGTGACGTCA	8	7 (88%)	7 (88%)	8 (100%)	7 (88%)	8 (100%)	1 (13%)
ETS⇔CRE	16-mer	CCGGAAGTGACGTCAC	3	2 (67%)	1 (33%)	2 (67%)	1 (33%)	3 (100%)	2 (67%)
ETS⇔CRE	16-mer	GCGGAAGTGACGTCAC	2	2 (100%)	2 (100%)	2 (100%)	2 (100%)	2 (100%)	0 (0%)
N_1_CGN_7_CG	12-mer	ACGCACACACCG	45	5 (11%)	1 (2%)	3 (7%)	1 (2%)	19 (42%)	18 (40%)
N_2_CGN_7_CG	13-mer	CACGCACACACCG	27	2 (7%)	1 (4%)	1 (4%)	0 (0%)	9 (33%)	9 (33%)

Two versions of the ETS motif that localize in proximal promoters differ only in the first nucleotide, the more common CCGGAA and the rarer GCGGAA (Figure S3A) (FitzGerald *et al.* 2004). DNA binding specificities of the 27 human ETS family members identify three proteins (SPI1, SPIB, and SPIC) that preferentially bind the rarer ETS motif (Kaplan *et al.* 2010). The rarer GCGGAA ETS motif is enriched compared with the CCGGAA motif in the ETS⇔CRE motif (Figure S3B).

### Molecular model of ETS⇔CRE motif bound by DNA

To evaluate the potential for simultaneous binding of three proteins (ETS monomer and CREB dimer) to the ETS⇔CRE motif, we built a molecular model using PDB files of the ETS1 protein bound to an ETS site (PDB ID: 1K79) (Garvie *et al.* 2001) and the CREB dimer bound to the CRE (PDB ID: 1DH3) (Schumacher *et al.* 2000). The two structures were aligned computationally after superimposing 10 DNA bases on each strand of DNA. The combined structure did not produce protein clashes, suggesting that both proteins could potentially bind the ETS⇔CRE motif simultaneously (Figure 3, C–E). The **GTG** trinucleotide, which is common to both the ETS and CRE motifs, interacts with both proteins in the model. The ETS domain, a winged helix-turn-helix protein fold, interacts with the major groove using an α-helix to bind the core GGAA of the motif. It also crosses the phosphate backbone and interacts with the minor groove of the **GTG** trinucleotide (Hollenhorst *et al.* 2011b). The CREB dimer interacts with the **GTG** trinucleotide in the major groove and never crosses the DNA backbone.

### The ETS protein GABPα and the B-ZIP protein CREB preferentially bind to ETS⇔CRE

**Figure 4:**
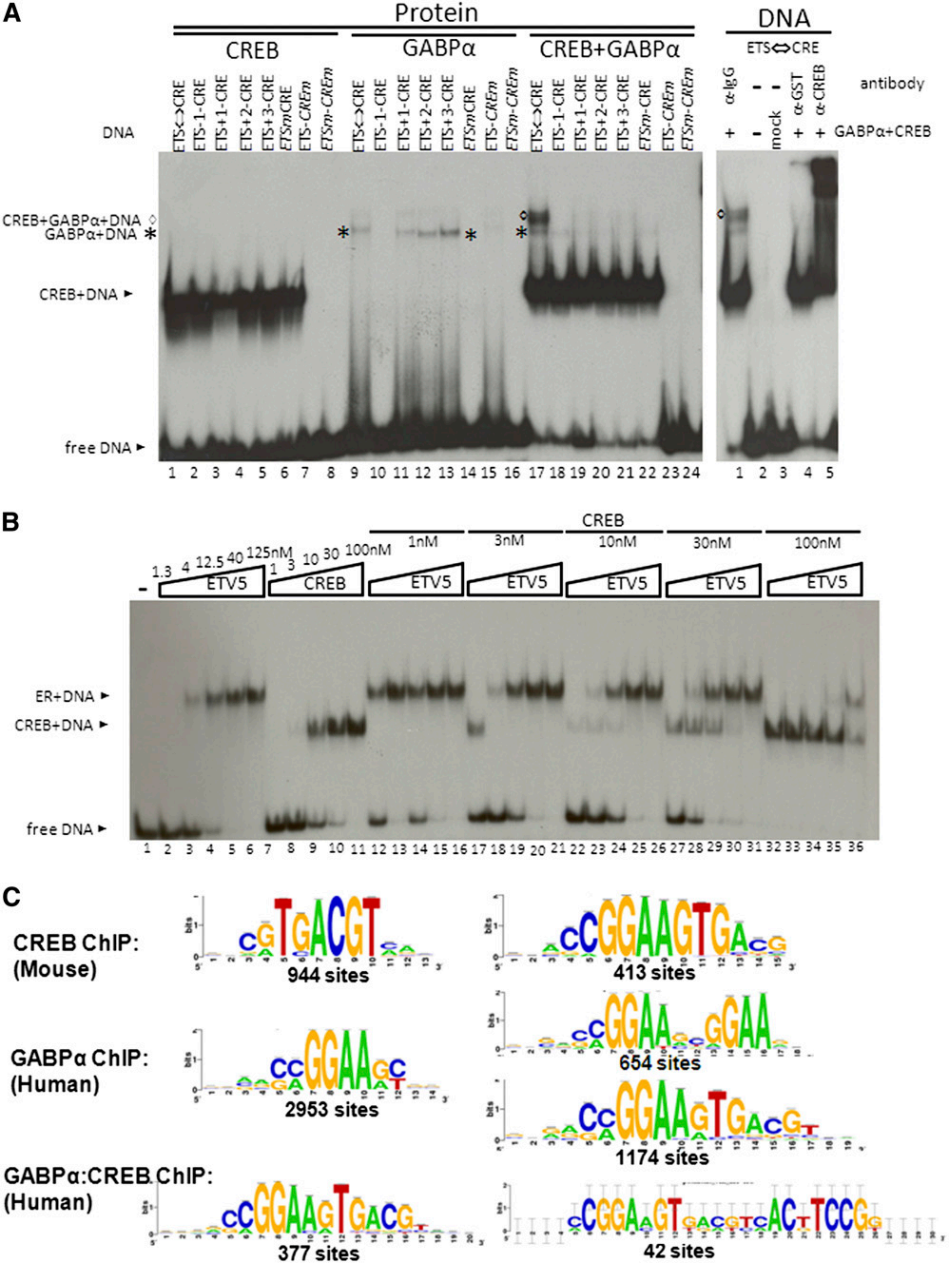
EMSA showing preferential DNA binding of the ETS protein GABPα and B-ZIP protein CREB to the ETS⇔CRE sequence (GCGGAA**GTG**ACGTCA). (A) Left panel: The DNA binding domain of GABPα with N-terminal GST tag and the B-ZIP domain of CREB were *in vitro* translated alone or together, and subjected to EMSA with eight DNA probes (Table 1). Lanes 1–8, 3 nM CREB; lanes 9–16, 200 nM GABPα; lanes 17–24, 3 nM CREB and 200 nM GABPα. Right panel: Supershift experiment demonstrates that the indicated CREB-GABPα-DNA complex contains both CREB and GABPα. Lanes 1, 4, and 5, *in vitro* translated 3 nM CREB and 200 nM GABPα; lane 2, no protein; lane 3, *in vitro* translation without protein-encoding DNA.*GABPα-DNA complex; ^◊^CREB-GABPα-DNA complex. (B) A dose response EMSA of the ETS protein ETV5 and B-ZIP protein CREB binding to the ETS⇔CRE sequence (GCGGAA**GTG**ACGTCA). Increasing concentrations of ETV5 (1.3, 4, 12.5, 40, and 125 nM) or CREB (1, 3, 10, 30, and 100 nM) alone shows dose-responsive binding (lanes 2–6 and lanes 7–11) to the ETS⇔CRE motif. Increasing concentrations of ETV5 with fixed concentrations of CREB shows that both proteins cannot simultaneously bind to the ETS⇔CRE motif. (C) Enriched motifs generated using the peak-motifs package of Regulatory Sequence Analysis Tools (RSAT). For *de novo* motif detection, we used all 6442 human GABPα ChIP-seq peaks (Valouev *et al.* 2008) and all 3998 mouse CREB ChIP-seq peaks (Martianov *et al.* 2010) as input sequences. In CREB ChIP-seq peaks, the most enriched motif is the canonical CRE, and ETS⇔CRE motif is among the other significantly enriched motifs. In GABPα ChIP-seq peaks, ETS motif is the primary enriched motif, and ETS⇔ETS is among the other enriched motifs. *De novo* motif detection using all 2953 ETS motif–containing regions predicted ETS⇔CRE as the best-enriched motif. *De novo* motif detection using 1453 commonly bound region by CREB and GABPα predicted ETS⇔CRE as the best-enriched motif. ETS⇔CRE⇔ETS is one of the other enriched motifs in these regions. The number of sites below each motif indicates the number of peaks that have at least one predicted motif.

EMSA was used to investigate whether ETS and B-ZIP proteins could simultaneously bind the ETS⇔CRE motif (Table 1). In the EMSA experiments, we used the B-ZIP protein CREB to bind the CRE motif and the ETS proteins GABPα or ETV5 to bind the ETS motif (Figure 4). Eight DNA probes were examined. Three DNA probes contained mutations in either or both motifs that abolished protein binding to the expected TFBS (Figure 4A). Five DNA probes examined the spacing between the two motifs; one probe has a deletion of 1-bp and three DNA probes have an insert of 1-bps, 2-bps, or 3-bps between the ETS and CRE motifs. CREB bound well at 10 nM (Ahn *et al.* 1998), whereas GABPα binding was weaker, being detectable at 200 nM. When GABPα and CREB were mixed, GABPα binding was enhanced only on the DNA probe containing the ETS⇔CRE motif (compare lane 17 with lane 9 of Figure 4A). None of the deletion or insertion probes form the CREB|GABPα|DNA complex (lanes 18–24, Figure 4A). Supershift experiments demonstrated that both GABPα and CREB proteins were present in the complex formed only on the ETS⇔CRE motif containing DNA probe (Figure 4A), suggesting that this specific overlap of three base pairs between ETS and CRE motifs is important for binding by both GABPα and CREB. Importantly, the ETV5 member of the ETS family formed neither the CREB|ETV5|DNA complexes nor the CREB|DNA or ETV5|DNA complex forms (Figure 4B). A dose-response EMSA showed that binding of one protein precludes the binding of another protein. Even when we saturated the probes with higher concentrations of ETV5 or CREB proteins, no CREB|ETV5|DNA complex was observed.

### Motif detection in CREB and GABPα ChIP-seq peaks

We examined published ChIP-seq data sets for GABPα (Valouev *et al.* 2008) in humans and CREB in mouse (Martianov *et al.* 2010) to determine whether the ETS⇔CRE motif is enriched in the ChIP-seq peaks. The peak-motif package (Thomas-Chollier *et al.* 2011) of RSAT was used for evaluating the enriched motifs in these ChIP-seq regions. Using all CREB peak regions, the peak-motif identified the overlapping ETS⇔CRE motif, which is more enriched than the canonical CRE motif (Figure 4C and Table 3). When we used only the GABPα ChIP-seq peaks for *de novo* motif detection, we identified the canonical ETS and the ETS⇔ETS motif, but not the ETS⇔CRE motif. However, when we examined the 2953 peaks that contain the canonical ETS motif, we detected that the ETS⇔CRE motif is the best-enriched motif (Figure 4C).

**Table 3:** Enrichment of ETS, CRE and ETS⇔CRE motifs in CREB and GABPα ChIP-seq peaks

Motifs	N-mers	DNA Sequence	Mouse Whole Genome (100%)	CREB ChIP-seq Peaks (%)	Human Whole Genome (100%)	GABPα ChIP-seq Peaks (%)
ETS	8-mer	CCGGAAGT	16,346	652 (4%)	14,031	1459 (10%)
CRE	8-mer	TGACGTCA	14,297	591 (4%)	10,389	180 (2%)
ETS⇔CRE	11-mer	CGGAAGTGACG	238	118 (50%)	226	179 (79%)
ETS⇔CRE	12-mer	CGGAAGTGACGT	89	51 (57%)	93	80 (86%)
ETS⇔CRE	12-mer	CGGAAGTGACGC	82	48 (59%)	81	67 (83%)
ETS⇔CRE	13-mer	CCGGAAGTGACGT	45	25 (56%)	35	35 (100%)
ETS⇔CRE	13-mer	GCGGAAGTGACGT	21	15 (71%)	32	28 (88%)
ETS⇔CRE	13-mer	CCGGAAGTGACGC	42	27 (64%)	52	42 (81%)
ETS⇔CRE	13-mer	GCGGAAGTGACGC	28	21 (75%)	19	17 (90%)
ETS⇔CRE	15-mer	CGGAAGTGACGTCA	12	8 (67%)	7	5 (71%)

An additional analysis used the GABPα ChIP-seq data already described from human Jurkat cell line and CREB ChIP-chip data from human HEK293T cells (Zhang *et al.* 2005). One thousand four hundred sixty-three (1463) peaks are common between CREB and GABPα binding sites. *De novo* motif detection using these regions by peak-motif detected ETS⇔CRE motif as the best-enriched motif (Figure 4C). Interestingly, among the other enriched motifs, we observed a palindromic ETS⇔CRE⇔ETS motif, in which the second ETS canonical motif is in the opposite strand (Figure 4C), suggesting the biological significance of the coordinated regulation of ETS and CREB in regulating the gene expression. The promoters with ETS⇔CRE, obtained from the commonly bound regions by CREB and GABPα, are significantly enriched for the GO terms of proteolysis involved in macromolecule catabolic process, RNA processing, and cellular response to stress (Table 4). However, the MEME-ChIP package (Machanick & Bailey 2011) of the MEME Suite failed to detect the ETS⇔CRE motif as an enriched motif in any data set.

**Table 4:** Enriched GO terms of genes commonly bound by CREB and GABPα with ETS⇔CRE motifs

Term	Name	Count	*P*	Corrected *P* (Benjamini)
GO:0006396	RNA processing	32	7.5E-09	9.8E-06
GO:0009057	Macromolecule catabolic process	31	4.2E-05	7.9E-03
GO:0033554	Cellular response to stress	25	5.7E-05	9.4E-03

### Length of ETS⇔CRE motif

Two strategies were used to evaluate the length of the ETS⇔CRE motif: (i) enrichment in 8000 housekeeping DNase I hypersensitive sites (DHS) (Sabo *et al.* 2004) and (ii) conservation in mammalian genomes.

**Figure 5:**
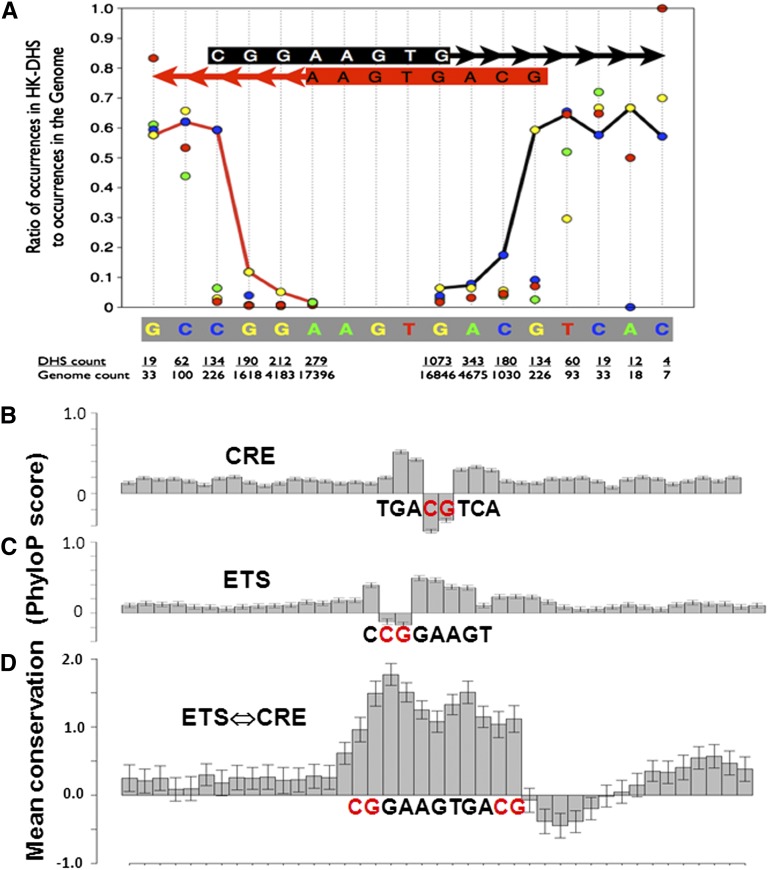
(A) Preferential localization in housekeeping DHS compared with the genome for different length of ETS⇔CRE sequences. The ETS (CGGAAGTG) and CRE (AAGTGACG) 8-mers were lengthened toward the indicated arrows, and for each bp extension, preferential localization in housekeeping DHS are calculated. A jump in localization of ETS (CGGAAGTG) occurs when the second CG dinucleotide is included, which creates the 11-mer CGGAAGTGACG. The ETS 8-mer CGGAAGTG occurs 16,846 times in the genome and 1073 times in housekeeping DHS, a ratio of ∼8%. The ratio in housekeeping DHS of 8-mers (CGGAAGTN) with a different final nucleotide are shown as a colored dot (G = yellow, A = green, T = red, C = blue). The ETS 9-mer CGGAAGTGA occurs 343 times in housekeeping DHS with a similar enrichment in housekeeping DHS as the 8-mer. When the sequence is extended to the 11-mer CGGAAGTGACG, enrichment in housekeeping DHS jumps to 60%. If the final G in the 11-mer is changed to the three other nucleotides, enrichment in housekeeping DHS is only 10%. When the ETS⇔CRE motif is extended to a 12-mer and beyond, enrichment in housekeeping DHS remains constant. When the ETS⇔CRE motif is extended from the CRE side toward the ETS side, a jump in localization in housekeeping DHS occurs when the AAGTGACG 8-mer is extended to the CGGAAGTGACG 11-mer. (B) Conservation or phyloP score in 30 mammals for the CRE 8-mer. (C) phyloP score for the ETS 8-mer. (D) phyloP score for the ETS⇔CRE 11-mer.

We extended the ETS motif 8-mer CGGAAGTG toward the CRE (Figure 5A) and counted the occurrences in the genome and known regulatory regions, including annotated promoters, proximal promoters, CpG islands, housekeeping DHS, and all DHS identified in 45 cell types (Sabo *et al.* 2004) (Table S2). The housekeeping DHS are defined as the DNase hypersensitive regions that are present in all 45 cell types (Sabo *et al.* 2004). The ETS 8-mer CGGAAGTG occurs 16,846 times in the genome and 6% of them are in housekeeping DHS. Similar results were observed when the motif is extended to the 9-mer (CGGAAGTGA) and 10-mer (CGGAAGTGAC). A transition occurs with the 11-mer (CGGAAGTGACG), with 60% occurring in housekeeping DHS and 83% occurring in known regulatory regions (Table S2). The 11-mer contains two CG dinucleotides, which are rare outside of regulatory regions.

It is important to note that the 11-mer CGGAAGTGACT can represent the overlapping of an ETS motif and an AP1 motif (TGA^C^/_G_TCA) to create the ETS⇔AP1. The ETS⇔AP1 motif may be cooperatively bound by an ETS protein and B-ZIP proteins that bind the AP1 motif. This sequence does not occur in housekeeping DHS, but it is enriched in tissue-specific DHS (Table 2) as observed previously (Hollenhorst *et al.* 2011b). When the motif is extended to a 12-mer, localization in housekeeping DHS does not increase but the occurrence decreases, indicating that the 11-mer is the core of longer and diverse ETS⇔CRE motifs (Figure 5A).

When the motif is extended from the CRE side toward the ETS motif, we again observe that localization in housekeeping DHS jumps to its maximal value when the motif is extended to the second CG and forms the 11-mer CGGAA**GTG**ACG. This suggests that the 226 ETS⇔CRE 11-mers in the genome contain different versions of the longer ETS⇔CRE 16-mers that may have distinct functions when they are bound by different combinations of ETS and B-ZIP family members.

### Conservation of the ETS⇔CRE motif in mammals

The conservation of the ETS⇔CRE motif was examined in 36 mammalian genomes (Pollard *et al.* 2010). Initially, we examined the PhyloP signature for the ETS (CGGAAGTG) and CRE (TGACGTCA) 8-mers. Both PhyloP signatures show conservation (Figure 5, B and C), except for the CG that has negative PhyloP values. We presume this simply reflects the chemical deamination of the C in the CG dinucleotide when it is methylated, a well-known hypermutable process that is not directly modeled in PhyloP. In contrast, in the ETS⇔CRE 11-mer (GGAA**GTG**ACG), all nucleotides, including both CG, are “highly” conserved, having scores four times larger than either the ETS or CRE motifs (Figure 5D). Conservation extends 1-bp beyond the CG on the ETS (5′) side of the motif to either a C or G, which is known to affect DNA binding of ETS family members (Wei *et al.* 2010). Beyond the CG on the CRE (3′) side to the ETS⇔CRE motif, the 4-bps (TCAC) region, which is the second half of the CRE motif, is not conserved. Provocatively, these nucleotides actually have negative PhyloP values and as here it does not have deamination effect of CG dinucleotides, it suggests that the sequences bound by the second monomer of the B-ZIP dimer in this context are evolving faster than neutral (Pollard *et al.* 2010).

### 1-bp variants of the ETS⇔CRE 11-mer

**Figure 6:**
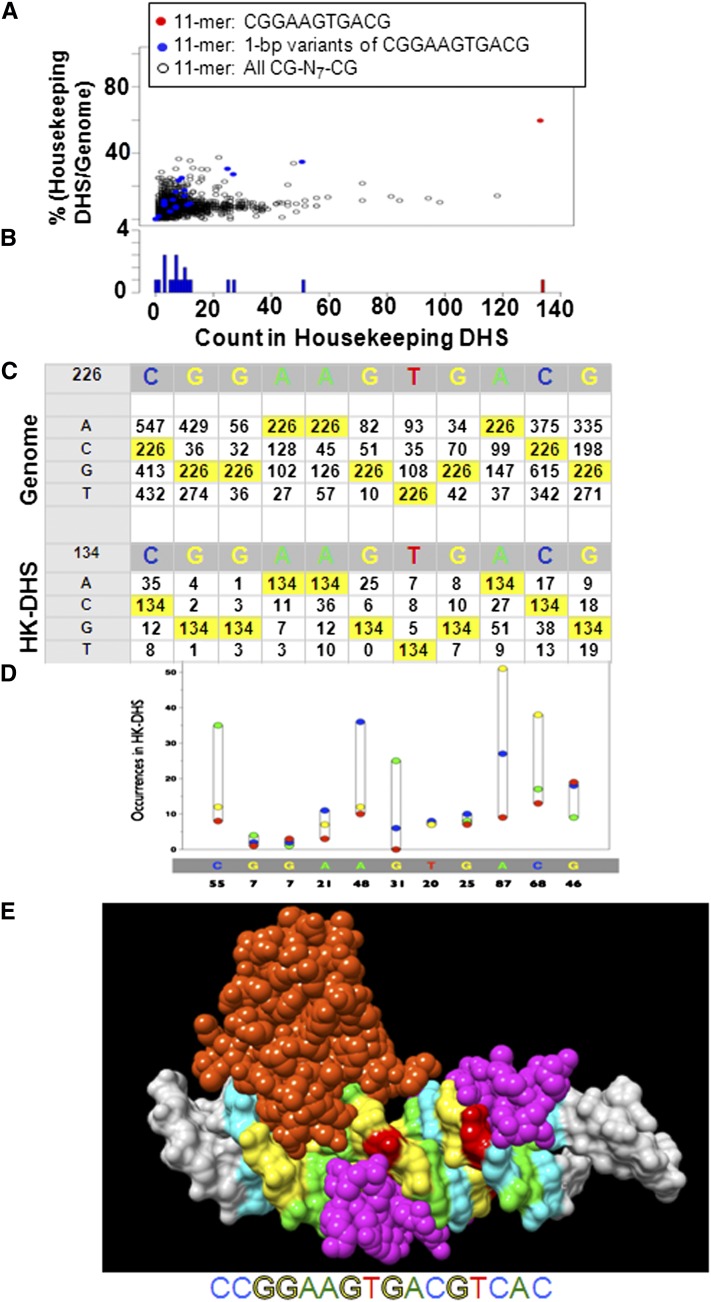
(A) Occurrences of the ETS⇔CRE 11-mer CGGAA**GTG**ACG and all 1-bp variants in housekeeping DHS *vs.* enrichment in housekeeping DHS compared with the genome. (B) Histogram showing abundance of the ETS⇔CRE 11-mer CGGAA**GTG**ACG and 1-bp variants in housekeeping DHS. (C) Table showing occurrences of 1-bp variances of the ETS⇔CRE 11-mer CGGAAGTGACG in the genome and housekeeping DHS. The numbers highlighted in yellow are the consensus ETS⇔CRE 11-mer. (D) Graphical presentation of the 1-bp variants of ETS⇔CRE 11-mer in housekeeping DHS. (E) Molecular model of the ETS⇔CRE motif with the nucleotides in color to highlight which parts of the structure are conserved and variable.

We examined whether 1-bp variants of the ETS⇔CRE 11-mer are also enriched in housekeeping DHS (Figure 6, A–D). Of the 147 occurrences, 51 (35%) of the most abundant 1-bp variant (CGGAAGTG*G*CG) are in housekeeping DHS. Two additional variants (CGGA*C*GTGACG and CGGAAGTG*C*CG) are abundant and enriched in housekeeping promoters, suggesting that they may also be functional. The GGA in the core of the ETS motif is critical for the sequence-specific binding (Graves & Petersen 1998) and shows very little variability in housekeeping DHS, suggesting that there are virtually no occurrences of the crippled ETS⇔CRE motif in regulatory regions. In the genome, all 1-bp variants that do not disrupt the CG are less abundant than the ETS⇔CRE 11-mer. In contrast, 1-bp variants that do disrupt either of the two CG are typically more abundant than the ETS⇔CRE, highlighting the profound effect of the CG dinucleotide on the occurrence of a DNA sequence in the genome. A molecular model of the ETS⇔CRE 16-mer bound by ETS and CREB is color-coded to visualize each nucleotide (Figure 6E). Potentially, the abundant 1-bp nucleotide variants of the ETS⇔CRE motif in housekeeping promoters are bound by different combinations of ETS and B-ZIP proteins.

### Four abundant ETS⇔CRE 13-mers **(^C^/_G_CGGAA**GTG**ACG^T^/_C_)**

**Figure 7:**
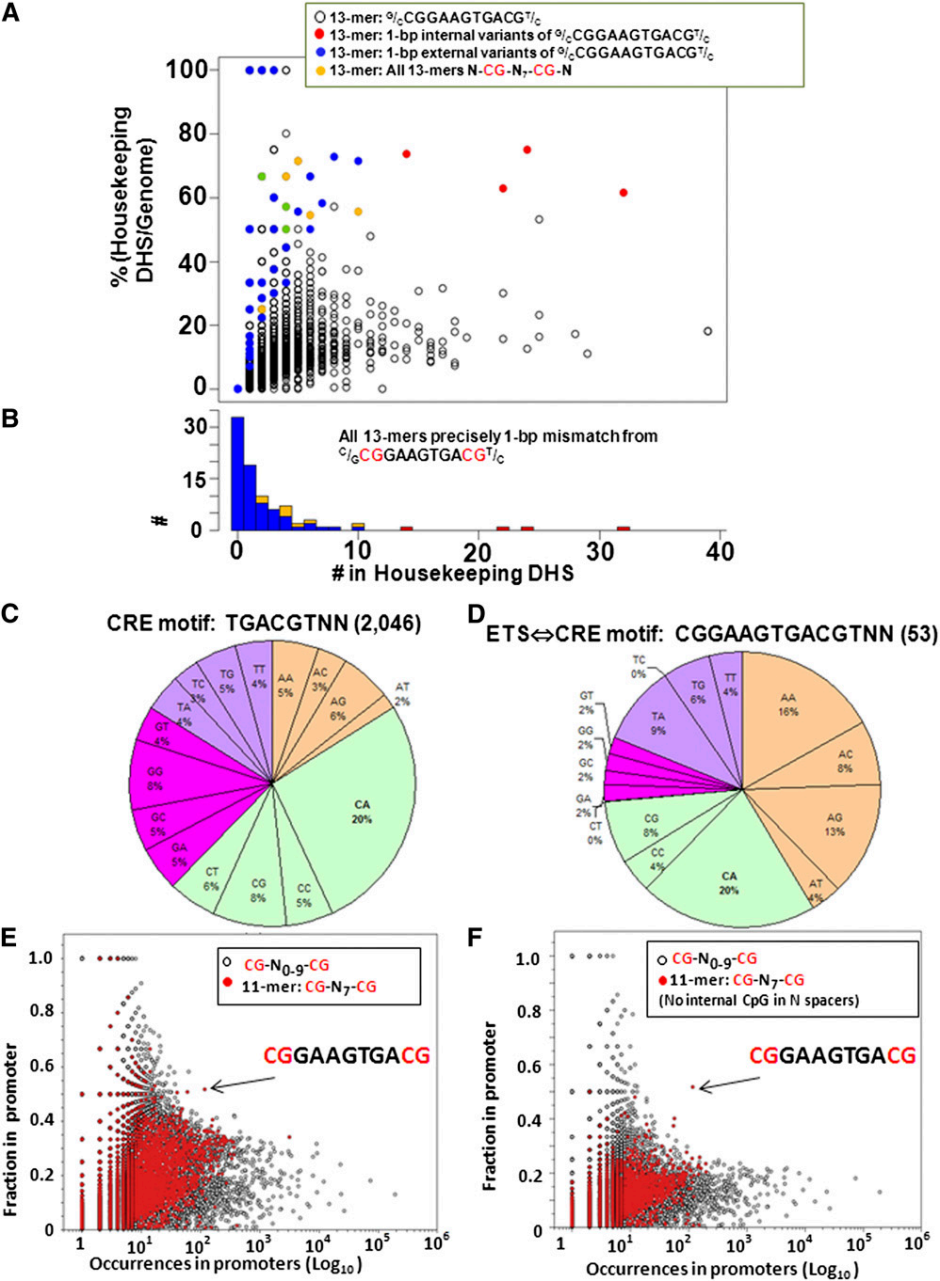
(A) Abundance of 4 ETS⇔CRE 13-mers (^C^/_G_CGGAA**GTG**ACG^T^/_C_) and 1-bp variants in housekeeping DHS *vs.* percentage of occurrences in housekeeping DHS compared with the genome. All N-CG-N_7_-CG-^13^N-mers are shown. The four abundant ETS⇔CRE 13-mers (^C^/_G_CGGAA**GTG**ACG^T^/_C_) are shown in red. (B) Histogram of occurrences of the ETS⇔CRE 13-mers ^C^/_G_CGGAA**GTG**ACG^T^/_C_ and all 1-bp variances in housekeeping DHS. (C) Pie chart representation of the occurrence of the dinucleotides at the end of the CRE motif TGACGTNN that occurs 2046 times in proximal promoters (−200-bps to +60-bps). (D) Pie chart representation of the occurrence of the dinucleotides at the end of the ETS⇔CRE motif CGGAAGTGACGTNN. (E) Preferential occurrence in promoters compared to the genome for all pairs of CG separated by 0-bps to 9-bps [CG-_(0-9)_-CG]. (F) Same as (E), but sequences with an internal CG are excluded. The one sequence that is abundant primarily in promoters is the ETS⇔CRE motif.

The abundance of longer versions of the ETS⇔CRE 11-mer in the genome and regulatory regions was evaluated (Figure S3C). We initially focused on 16-mers, the potential length of the ETS⇔CRE motif. Of the 226 11-mers in the genome, 171 different 16-mers occur, and the most abundant 16-mer (CCGGAAGTGACGCGAG) occurs seven times. The canonical motif CCGGAA**GTG**ACGTCAC occurs three times in the genome. The alignment of ETS⇔CRE 11-mers, including surrounding DNA sequences, identified four abundant ETS⇔CRE 13-mers (^C^/_G_CGGAA**GTG**ACG^T^/_C_) (Figure S3C), representing 70% of all ETS⇔CRE 11-mers (Figure 7, A and B). Each 13-mer correlated with different GO terms, suggesting distinct functions (Table S3). The nucleotide before the CG in the ETS motif is either G or C, and these are known to be bound by different ETS family members (Wei *et al.* 2010). The nucleotide after the central CG in the CRE is typically a pyrimidine, T and C. They are 5-fold more abundant than the purines G and A (Table 2). The T and C in this position are optimal for binding the B-ZIP proteins CREB and C/EBP, respectively (Johnson 1993). Each of the four ETS⇔CRE 13-mers is expected to be optimally bound by a specific combination of ETS monomers and B-ZIP dimers.

The dinucleotides following the CRE 6-mer TGACGT-N_2_ in proximal promoters are enriched only for CA dinucleotide, which produces the canonical CRE 8-mer TGACGTCA (Figure 7C). In contrast, the dinucleotides following the ETS⇔CRE 12-mer CGGAAGTGACGT are also enriched for AN dinucleotides, suggesting that the CRE and the ETS⇔CRE motifs in promoters are bound by different B-ZIP proteins (Figure 7D).

### Localization of pairs of CG in DHS

In the ETS⇔CRE motif, the two CG are separated by 7-bps (CG-N_7_-CG). To identify whether additional pairs of CG preferentially occur in promoters, we counted in the whole genome the occurrence of sequences containing a pair of CG separated by 0-bps to 9-bps (CG-N_0-9_-CG) and determined what fraction are in housekeeping DHS. The ETS⇔CRE motif stands out among all other sequences containing pairs of CG, being abundant and primarily in promoters (Figure 7, E and F).

### CG methylation status of the ETS⇔CRE motif in two mouse primary cells

Methylation of the CG dinucleotide in canonical ETS and CRE motifs inhibits binding of both ETS and CREB proteins (Iguchi-Ariga & Schaffner 1989; Umezawa *et al.* 1997; Rozenberg *et al.* 2008). An important feature of the ETS⇔CRE motif is the presence of two CG that can be methylated. We used two mouse methylomes at 100X coverage for newborn mouse dermal fibroblasts and 45X coverage for primary keratinocytes. The four ETS⇔CRE 13-mers have different methylation properties (Table S4, Figure S4, Figure S5, and Figure S6). All 21 occurrences of the GCGGAAGTGACGT 13-mer are unmethylated on both CG dinucleotides in dermal fibroblasts and keratinocytes, suggesting that they are in functional regions of the genome. Of the 45 occurrences of the more abundant 13-mer CCGGAAGTGACGT, 33 are unmethylated in both cells (Figure S4C, Figure S5A, and Figure S6A).

Not all 13-mers with two CG dinucleotides separated by 7-bp are unmethylated. Only 10% of CACGCACACACCG is unmethylated (Figure S4G, Figures 5E and 6E). Comparing two methylome data for these motifs shows that unmethylated 13-mer motifs are common and generally unmethylated in both cell types (Figure S6, A–D) and that these unmethylated ETS⇔CRE sequences are mainly enriched in promoters (Table S4), lending support to the suggestion that every occurrence of an unmethylated version of the ETS⇔CRE motif is biologically important.

## Discussion

We determined the distribution in human promoters of split DNA 8-mers consisting of a pair of 4-mers separated by 1-bp to 30-bps. A striking result is that few split 8-mers with insert length of 5-bps or greater (X_4_-N_5-30_-X_4_) localize in proximal promoters. This is in sharp contrast to Drosophila promoters, in which many split 8-mers with a 20-bp to 30-bp insert length (X_4_-N_20-30_-X_4_) localize in proximal promoters (Vinson *et al.* 2011). We examined split 8-mers in human promoters and identified pairs of 4-mers that localized at a specific insert length and not others. This article focused on the ETS motif (^C^/_G_CGGAA**GTG)** precisely overlapping with a CRE motif (**GTG**ACGTCAC) to create a composite site, the ETS⇔CRE motif (^C^/_G_CGGAA**GTG**ACGTCAC). The trinucleotide **GTG** is common in the two TFBS, being the 3′ end of the ETS motif and 5′ end of the palindromic CRE motif. Molecular modeling using X-ray structures of ETS and B-ZIP proteins binding the ETS⇔CRE motif suggests that the ETS monomer and B-ZIP dimer can bind the overlapping TFBS without any protein-protein clashes. Instead of ETS and B-ZIP proteins competing for binding the ETS⇔CRE motif, the ETS protein GABPα and the B-ZIP protein CREB preferentially bind the ETS⇔CRE motif only when the **GTG** trinucleotide overlaps. In contrast, the ETS protein ETV5 competes with CREB to bind the ETS⇔CRE motif. *De novo* enriched motif detection using the *in vivo* CREB and GABPα ChIP-seq binding regions identified the ETS⇔CRE motif along with the canonical CRE and ETS motifs, suggesting an *in vivo* function for the motif. Additionally, the conservation of the ETS⇔CRE motif is signifying its biological function (Xie *et al.* 2005; Pollard *et al.* 2010).

The ETS domain has been shown to interact with several different DNA binding proteins to bind sequences containing chimeric aspects of each TFBS (Hollenhorst *et al.* 2011b). The ETS protein GABPα initially was observed interacting with GABPβ to bind a chimeric sequence (Batchelor *et al.* 1998). ETS was subsequently shown to interact with additional proteins. The forkhead proteins interact at the 5′ end of the ETS motif (De Val *et al.* 2008), whereas SRF, PAX, and potentially CREB interact at the 3′ end of the ETS motif (Hollenhorst *et al.* 2011b). Several of these interactions have been identified by examining tissue-specific enhancer sequences (Hollenhorst *et al.* 2011b). The cytokine, RANTES (regulated upon activation, normal T cell expressed) is induced by LPS through binding in promoters by ATF and Jun proteins to a composite site containing non-overlapping ETS and CRE motifs (Boehlk *et al.* 2000).

ETS and CRE motifs co-occur in proximal promoters (FitzGerald *et al.* 2004). Cooperative DNA binding by GABPα and CREB to adjacent ETS and CRE sites separated by various distances up to 15-bps has been reported (Sawada *et al.* 1999). The cooperative binding is mapped to the non-DNA binding region of GABPα, suggesting that cooperativity is via protein-protein interactions. These investigators did not observe that the two motifs needed to be precisely aligned relative to each other for cooperative binding. These results are in sharp contrast to what we observed; the precise overlap produces enhanced GABPα and CREB binding, suggesting that the cooperative binding we observed between the ETS and CREB DNA binding domains is distinct from the cooperative binding observed when full-length proteins are examined. The ETS and CRE motifs at different spacing than the observed ETS⇔CRE motif may be preferentially bound by different combinations of ETS and B-ZIP proteins and may have specific functions in regulating gene expression. Oncogenic ETS family members in prostate cancer localize at ETS⇔AP1 motifs that have the same overlap (Hollenhorst *et al.* 2011a) observed in the ETS⇔CRE motif. The AP1 or TRE 7-mer (TGA^C^/_G_TCA) is a 1-bp deletion at the center of the CRE, disrupting the CG dinucleotide. Recently, the ETS and CRE motifs were observed to co-occur in ChIP-seq data sets with a spacing of 1-bp to 2-bp (Whitington *et al.* 2011), whereas we highlight the ETS⇔CRE motif at a precise spacing with unique biochemical properties.

Overlapping protein binding is observed in the enhanceosome where the ATF-2/c-Jun heterodimer binds to the same DNA base pairs as the IRF-3 protein. Again, there are no protein-protein interactions (Panne *et al.* 2004, 2007; Panne 2008); instead, it appears that the cooperative binding of these three polypeptides is via allosteric changes to the DNA. This is similar to what may occur when GABPα and CREB preferentially bind the ETS⇔CRE motif.

Recently, it was suggested that a fundamental difference between prokaryotic and eukaryotic systems is that eukaryotic systems have short TFBS that proteins do not recognize with sufficient specificity to bind to cognate sites exclusively (Wunderlich & Mirny 2009) and need to cooperate with other TF to displace a nucleosome and become functional (Polach & Widom 1996; Mirny 2010). The overlap of two TFBS as observed in the ETS⇔CRE motif creates a long DNA sequences that are generally rare in mammalian genomes and could thus function like a prokaryotic system in which each occurrence is functional.

An alternative method to create specificity in vertebrate genomes is to have two TFBS that only need to be within 150-bps of each other and function together because they compete with nucleosomes for binding (Polach & Widom 1996; Mirny 2010; Biddie *et al.* 2011). It appears that both mechanisms operate in mammalian genomes. An advantage of the overlapping TFBS is that it allows for cooperative binding between specific members of each TF family, thus increasing specificity. This is absent in the model of two TF independently binding to DNA to displace a nucleosome. The nucleosome displacement mechanism allows different TF to act cooperatively, and it allows selection of which family member is functioning.

We have taken a DNA-centric perspective to evaluate which DNA sequences are important, eschewing the common practice embodied in the use of position weight matrices (PWM), of averaging two or more DNA sequences to create a logo or hybrid sequence. An inherent issue with the DNA-centric perspective is to know the length of the DNA sequence. An upper bound to the length of a DNA sequence is when it becomes unique in the genome, instead of having thousands of occurrences in which only a subset is functional. Vertebrate genomes are not big enough to accommodate all possible 16-mers. The ETS⇔CRE 16-mer is long enough so that random occurrences are not expected. Here, we have taken the approach that different sequences should not be averaged because this could obscure details concerning longer sequences having a distinct function. For example, the ETS⇔CRE 13-mers GCGGAAGTGACGT and CCGGAAGTGACGT enrich for distinct GO terms in addition to having distinct methylation properties. Closer examination of proximal promoters may identify additional examples of pairs of DNA sequences that are constrained relative to each other as we observed for the ETS⇔CRE motif. The identification of these sequences will be essential as we deconvolute the genome into functional units.

## Supplementary Material

Supporting Information
