# Supplementary material for: Overlapping ETS and CRE Motifs (G/CCGGAAGTGACGTCA) Preferentially Bound by GABPα and CREB Proteins
Source: G3 (Bethesda). 2012 Oct 1;2(10):1243–56. doi: 10.1534/g3.112.004002 (PMC3464117; doi:10.1534/g3.112.004002)
Supplement: Supporting Information [file supp_2_10_1243__index.html]

Supporting Information 

# Overlapping ETS and CRE Motifs (G/CCGGAAGTGACGTCA) Preferentially Bound by GABPα and CREB Proteins

## Supporting Information for Chatterjee *et al.*, 2012

**Files in this Data Supplement:**

- Supporting Information - Figures S1-S6 and Tables S1-S4 (PDF, 1.8 MB)
- Table S4 - Occurrence of unmethylated versions of the ETS⇔CRE motifs in the mouse genome with 24,273 promoters and proximal promoters and 16,026 CpG Islands (PDF, 90 KB)
- Figure S1 - Localization of 8-mers in human promoters (PDF, 140 KB)
- Figure S2 - A) Distribution of the CRE 8-mer TGACGTCA in human promoters. B) LF for CRE 8-mer with insert length ranging from 0-bps to 30-bps (TGAC-N0-30-GTCA). C) Distribution of the ETS TFBS (CCGGAAGT) in promoters counting occurrence in 20 bp bins. D) LF for ETS 8-mer (CCGG-N0-30-AAGT) with insert length ranging from 0-bps to 30-bps (PDF, 328 KB)
- Figure S3 - A) Distribution of the two ETS TFBS (CCGGAA and GCGGAA). B) Distribution of the two split 8-mers GCGG-N5-ACGT and CCGG-N5-ACGT representing the ETS⇔CRE motif. C) Color representation of the sequences surrounding the 134 ETS⇔CRE 11-mers that occur in housekeeping DHSs with C=blue, G=yellow, A=green and T=red (PDF, 701 KB)
- Figure S4 - A) Occurrence in mouse promoters compared to the genome of all split 8-mer containing two CGs separated by 4-bps (N-CG-N4-CG-N3) as is observed in the ETS⇔ETS motif which is labeled. B) Occurrence in mouse promoters compared to the genome of all split 8-mer containing two CGs separated by 7-bps CG-N7-CG-N) as is observed in the ETS⇔CRE motif which is labeled C-F) Methylation status in mouse dermal fibroblasts of the 4 ETS⇔CRE 13-mers C/GCGGAAGTGACGT/C. Percent methylations of 1st and 2nd CpGs are plotted for each 4 motifs. The majority of occurrences have no CpG methylation on either CpG. G) Methylation of 1st and 2nd CGs for the 13-mer CACGCACACACCG with pairs of CpG separated by 7-bps showing both the CpGs in the motif are mostly methylated in dermal fibroblasts (PDF, 320 KB)
- Figure S5 - A-D) Methylation status in mouse primary keratinocytes of the 4 ETS⇔CRE 13-mers C/GCGGAAGTGACGT/C (PDF, 227 KB)
- Figure S6 - A-D) Comparison in methylation status of both the CpGs in 4 ETS⇔CRE 13-mers C/GCGGAAGTGACGT/C in mouse dermal fibroblasts and keratinocytes. E) Methylation of 1st and 2nd CGs for the 13-mer CACGCACACACCG in primary dermal fibroblasts and keratinocytes.(PDF, 253 KB)
- Table S1 - The Localization Factor (LF) for all continuous (X4-N0-X4) and split 8-mers (X4-N1-30-X4) was determined (PDF, 96 KB)
- Table S2 - Occurrence of different length of ETS⇔CRE motifs in the human genome, promoters, proximal promoters, CpG Islands and housekeeping DNAse hypersensitive sites (PDF, 92 KB)
- Table S3 - Enriched GO terms (P<0.05) for the human genes that have one of the 4 ETS⇔CRE 12-mer or 13-mers (C/GCGGAAGTGACGT/C) in promoters (PDF, 102 KB)
