# Supplementary material for: Overlapping ETS and CRE Motifs (G/CCGGAAGTGACGTCA) Preferentially Bound by GABPα and CREB Proteins
Source: G3 (Bethesda). 2012 Oct 1;2(10):1243–56. doi: 10.1534/g3.112.004002 (PMC3464117; doi:10.1534/g3.112.004002)
Supplement: Supporting Information [file supp_2.10.1243_004002SI.pdf]

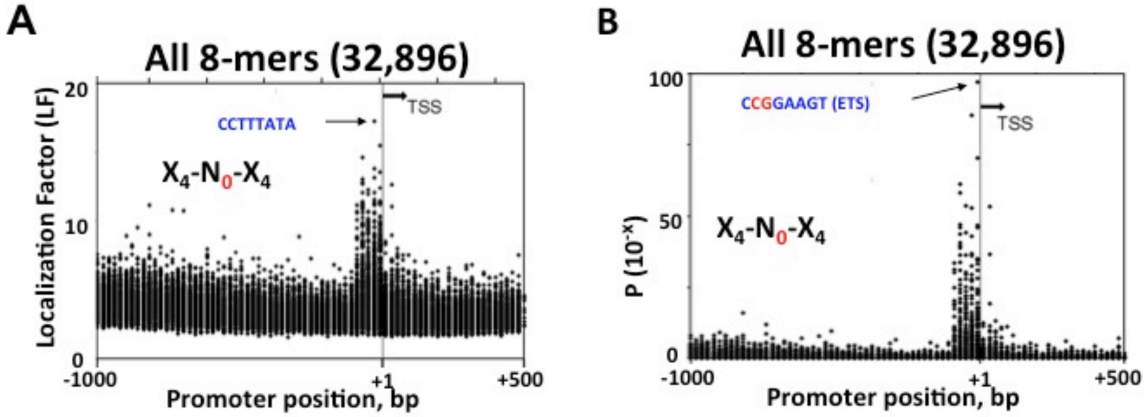

Figure S1 Localization of 8-mers in human promoters. **A)** Localization Factor (LF), a measure of non-random distribution of a DNA sequence, for 32,896 continuous 8-mers (X_4-N_0-X_4). For each 8-mer, the distribution in 17,143 human promoters (-1,000 bp to +500 bp) aligned relative to the TSS was determined and plotted in the most abundant 20 bp bin. Some sequences are preferentially localized near the TSS. **B)** Probability ($p=10^{-x}$) that an 8-mer have a non-random distribution is plotted in the most abundant bin for all 8-mers.

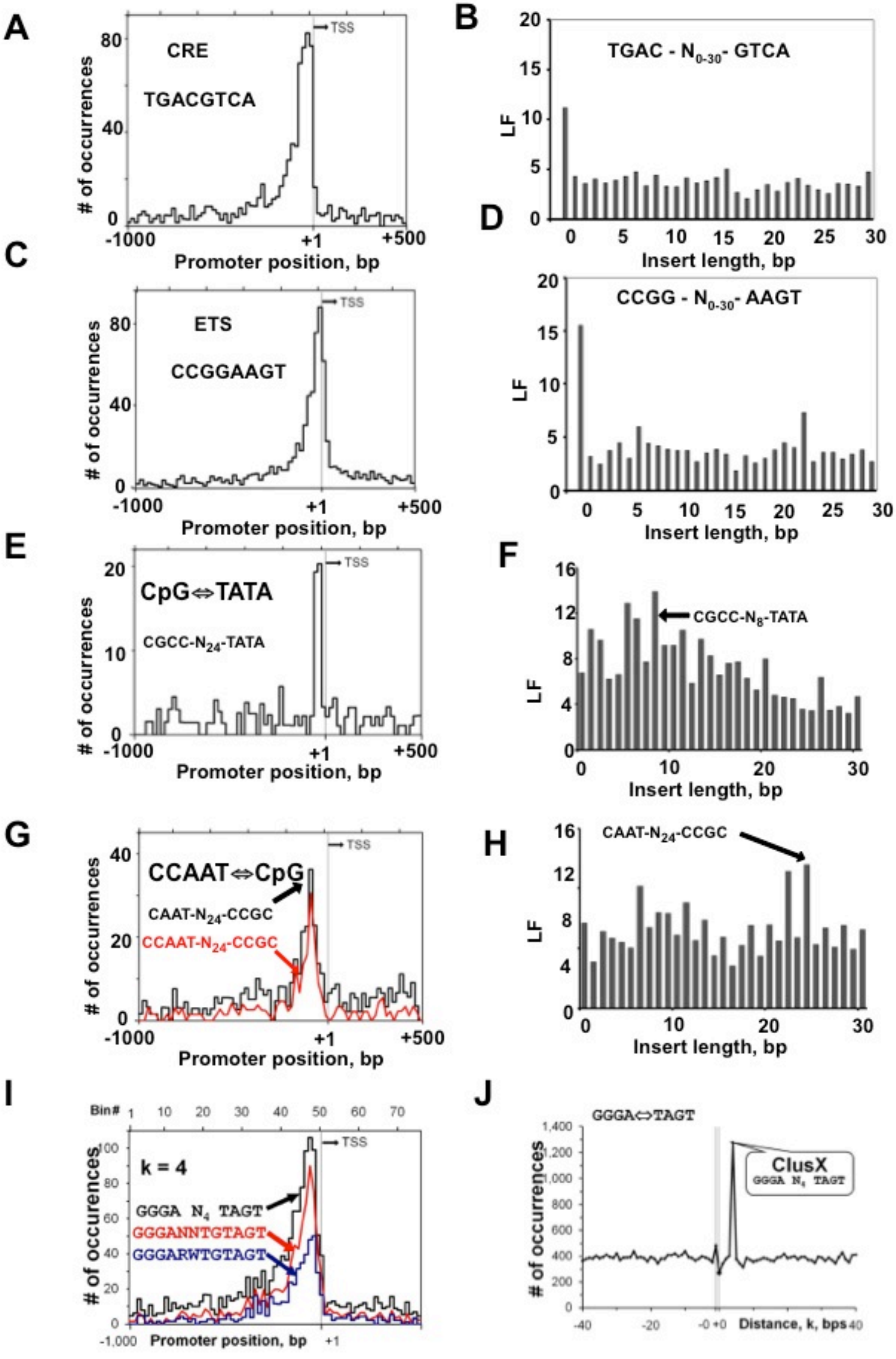

Figure S2 **A)** Distribution of the CRE 8-mer TGACGTCA in human promoters. **B)** LF for CRE 8-mer with insert length ranging from 0-bps to 30-bps (TGAC-$N_{0\text{-}30}$-GTCA). **C)** Distribution of the ETS TFBS (CCGGAAGT) in promoters counting occurrence in 20 bp bins. **D)** LF for ETS 8-mer (CCGG-$N_{0\text{-}30}$-AAGT) with insert length ranging from 0-bps to 30-bps. The 8-mer CCGGAAGT preferentially localize in promoters when the two 4-mers are abutted. High LF values are also observed at additional insert

length, including CCGG-N_6-AAGT, which represents the ETS⇔ETS motif and CCGG-N_{23}-AAGT. **E)** Distribution of the split 8-mer CGCC-N_{24}-TATA in promoters. **F)** LF for CGCC-$N_{0\text{-}30}$-TATA with insert length ranging from 0-bps to 30-bps. **G)** Distribution of the split 8-mer CAAT-N_{24}-CCGC in human promoters. The most localizing motif is the split 9-mer CCAAT-N_{24}-CCGC marked in red. **H)** LF for CAAT-$N_{0\text{-}30}$-CCGC with insert length from 0-bps to 30-bps. **I)** Distribution of the split 8-mer GGGA-N_4-TAGT and more localizing split 10-mer GGGA-N_2-TGTAGT. **J)** Occurrences of split 8-mer GGGA-$N_{0\text{-}40}$-TAGT with insert length ranging from 0-bps to 40-bps. The reverse order of the two 4-mers TAGT-$N_{0\text{-}40}$-GGGA is shown with negative values.

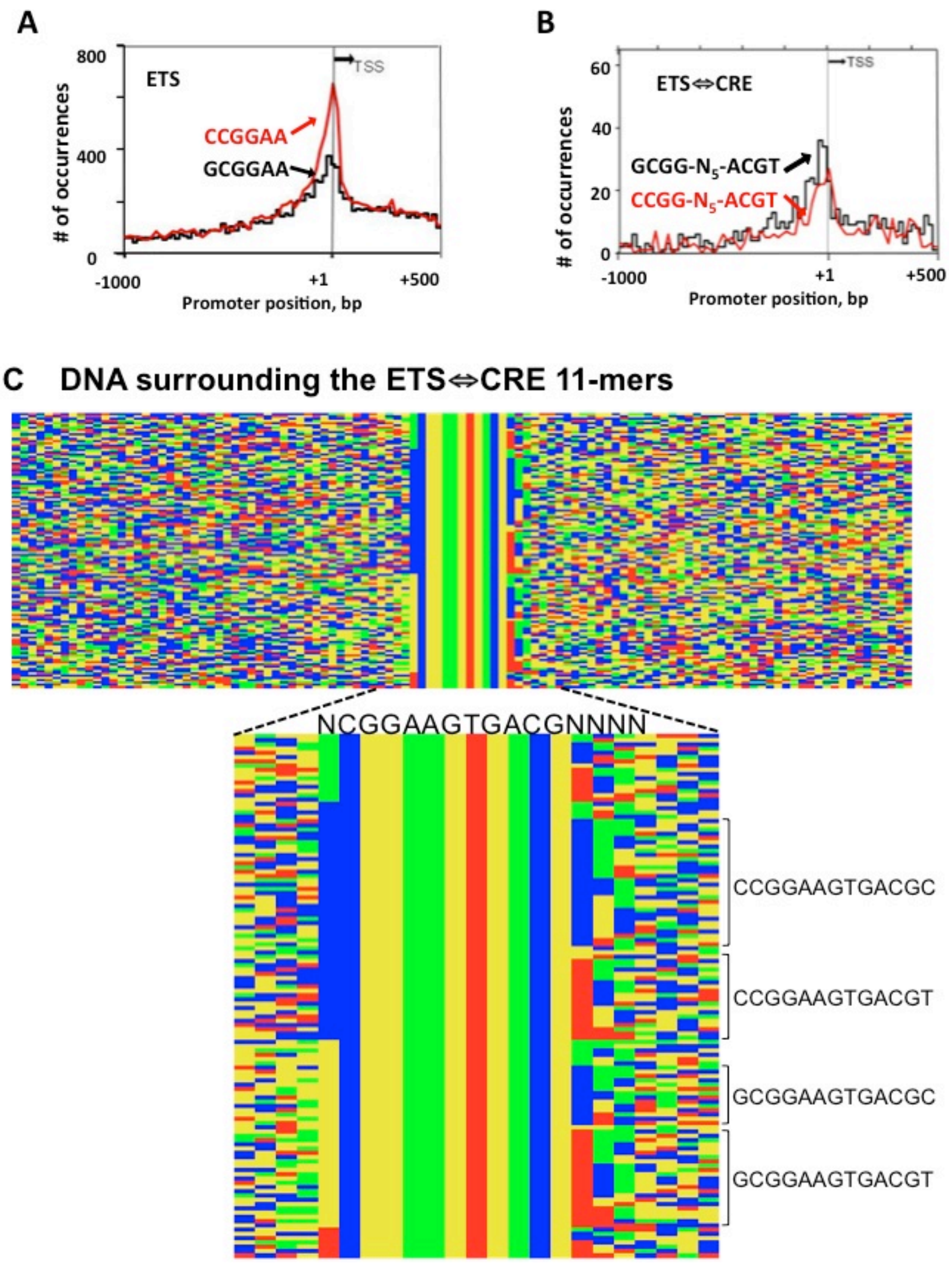

Figure S3 **A)** Distribution of the two ETS TFBS (CCGGAA and GCGGAA). **B)** Distribution of the two split 8-mers GCGG-N_5-ACGT and CCGG-N_5-ACGT representing the ETS⇔CRE motif. **C**) Color representation of the sequences surrounding the 134 ETS⇔CRE 11-mers that occur in housekeeping DHSs with C=blue, G=yellow, A=green and T=red. The core part of the ETS⇔CRE motif is shown in inset. Sequences were grouped based on the nucleotides as shown by numbers in bold: **1**CGGAAGTGACG**2345** where the numbers represent the order of grouping.

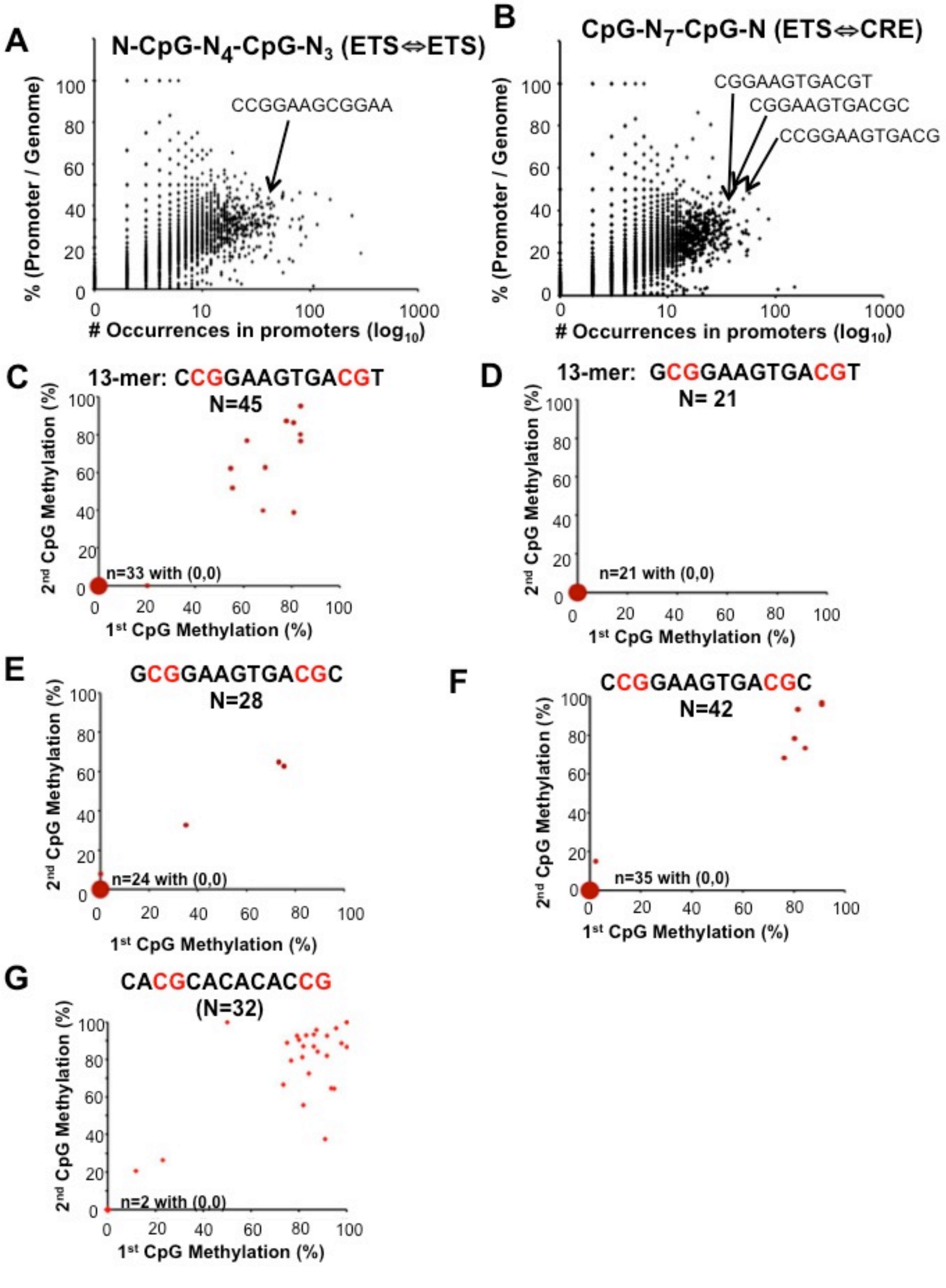

Figure S4 **A)** Occurrence in mouse promoters compared to the genome of all split 8-mer containing two CGs separated by 4-bps (N-CG-N_4-CG-N_3) as is observed in the ETS⇔ETS motif which is labeled. **B)** Occurrence in mouse promoters compared to the genome of all split 8-mer containing two CGs separated by 7-bps CG-N_7-CG-N) as is observed in the ETS⇔CRE motif which is labeled **C-F)** Methylation status in mouse dermal fibroblasts of the 4 ETS⇔CRE 13-mers $^{C}/_{G}$CGGAA**GTG**ACG$^{T}/_{C}$. Percent methylations of 1st and 2nd CpGs are plotted for each 4 motifs. The majority of occurrences have no CpG methylation on either CpG. **G)** Methylation of 1st and 2nd CGs for the 13-mer CACGCACACACCG with pairs of CpG separated by 7-bps showing both the CpGs in the motif are mostly methylated in dermal fibroblasts.

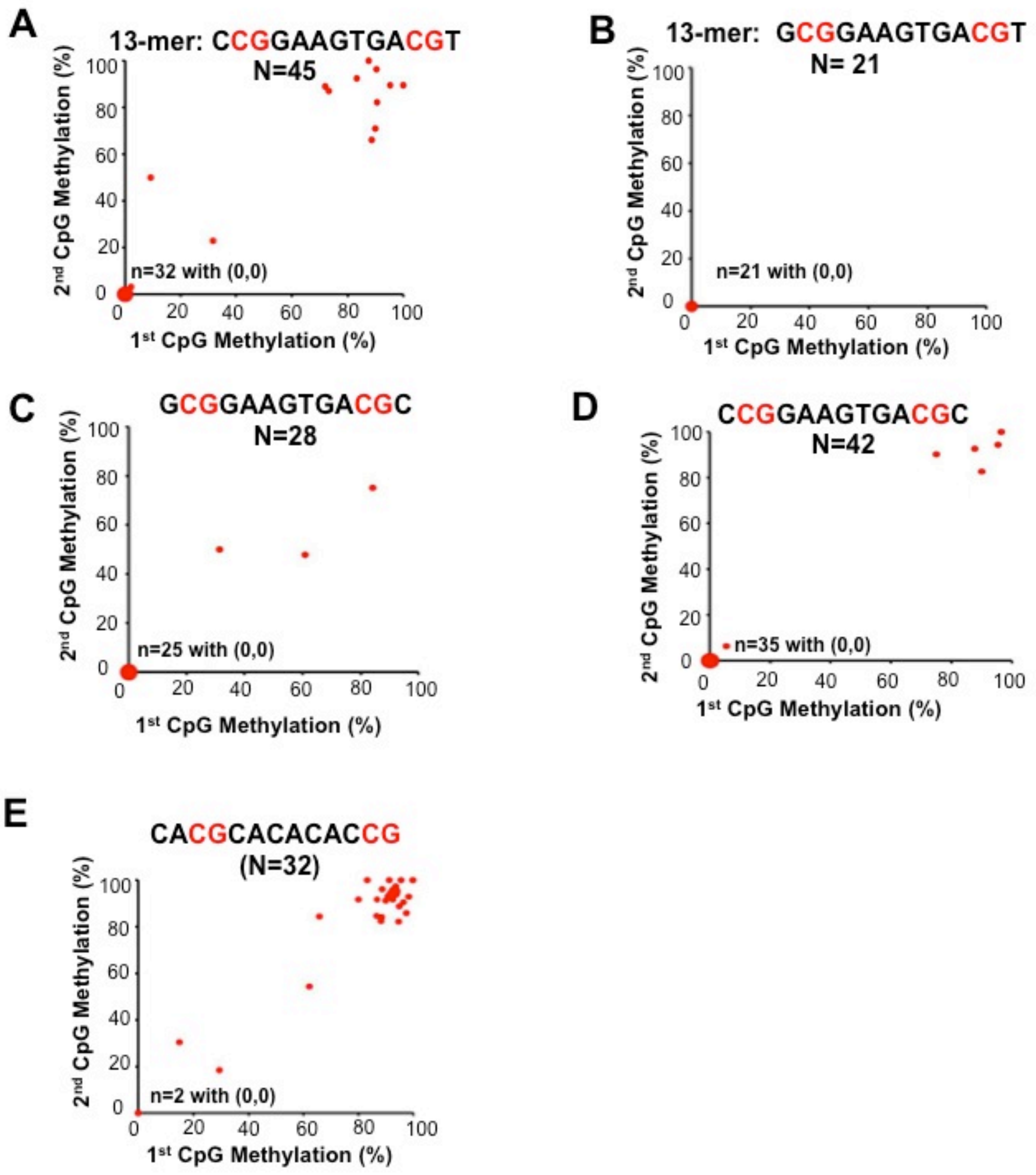

Figure S5 A-D) Methylation status in mouse primary keratinocytes of the 4 ETS⇔CRE 13-mers $^{C}/_{G}$CGGAA**GTG**ACG$^{T}/_{C}$. Percent methylations of 1st and 2nd CpGs are plotted. The majority of occurrences have no CpG methylation on either CpG. **E)** Methylation of 1st and 2nd CGs for the 13-mer CACGCACACACCG with pairs of CpG separated by 7-bps showing both the CpGs in the motif are mostly methylated in keratinocytes.

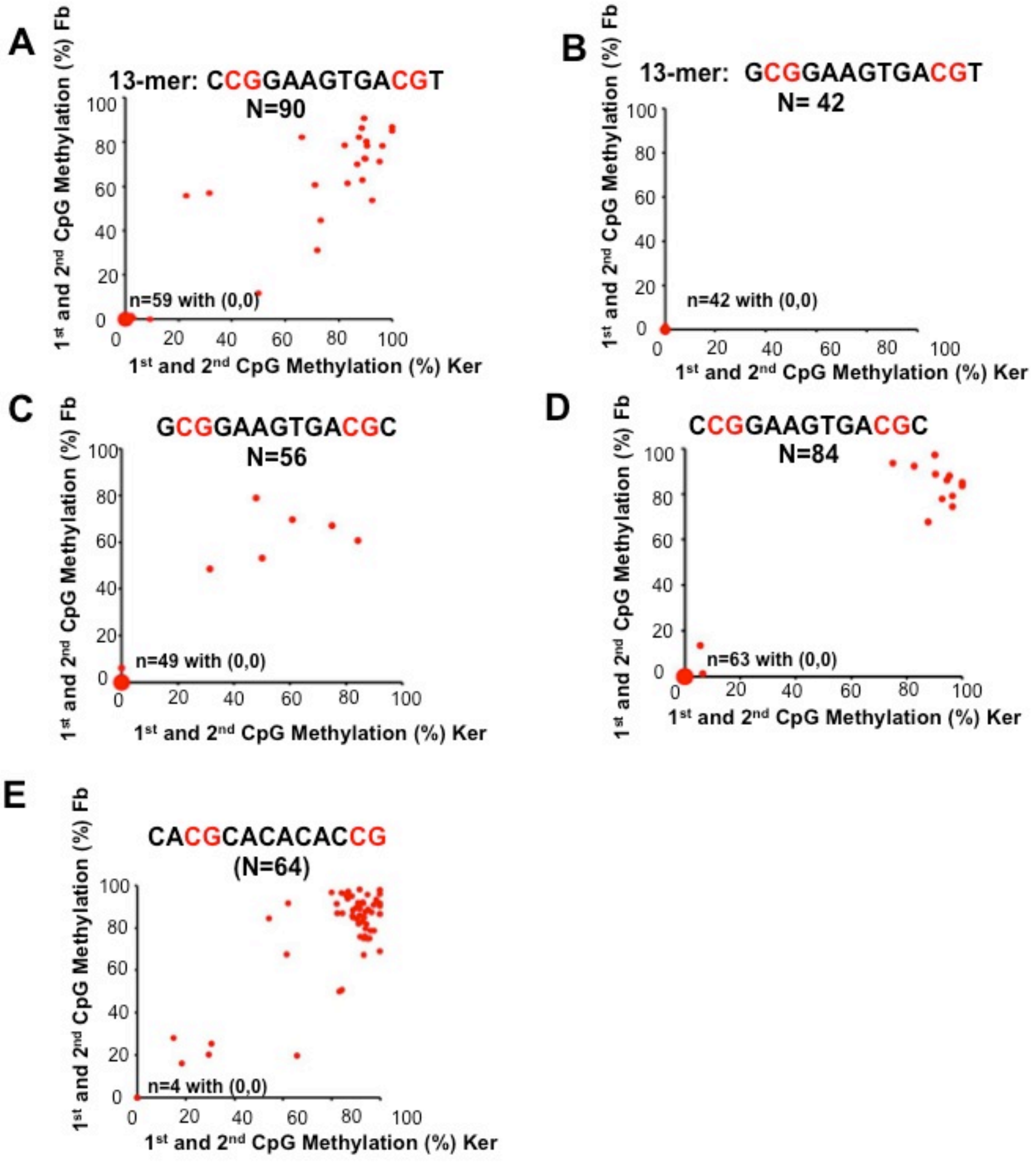

Figure S6 A-D) Comparison in methylation status of both the CpGs in 4 ETS⇔CRE 13-mers $^{C}/_{G}$CGGAA**GTG**ACG$^{T}/_{C}$ in mouse dermal fibroblasts and keratinocytes. **E)** Methylation of 1st and 2nd CGs for the 13-mer CACGCACACACCG in primary dermal fibroblasts and keratinocytes.

Table S1 The Localization Factor (LF) for all continuous (X_4-N_0-X_4) and split 8-mers (X_4-$N_{1\text{-}30}$-X_4) was determined. Next, the probability of a non-random distribution was determined and the top 20 for different insert length was determined. For column X_4-$N_{5\text{-}30}$-X_4, the insert length that produced the highest LF is presented. The most localized split 8-mers are further extended to determine the TFBS or overlapping TFBS and are presented in the column next to the sequence motifs. Two sequences representing the ETS⇔ETS motif and the ETS⇔CRE motif are highlighted in red.

X_4-N_0-X_4			X_4-N_2-X_4			X_4-N_4-X_4			X_4-$N_{5\text{-}30}$-X_4		
**Sequence**	**Predicted TFBS**	**P-value**	**Sequence**	**Predicted TFBS**	**P-value**	**Sequence**	**Predicted TFBS**	**P-value**	**Sequence**	**Predicted TFBS**	**P-value**
CCGGAAGT	ETS	97	GTGA--TCAC	CRE	56	TATA----CGGC	CpG-TATA	92	ACGT- N_9-GCGC	CpG-CRE	56
TATAAAGG	TATA	85	CGGA--TGAC	**ETS-CRE**	47	TATA----CGGG	CpG-TATA	36	GCGC-N_7-TGAC	CpG-CRE	44
CGGAAGTG	ETS	70	GCCC--CCCC	SP1	47	CAAT----GCGC	CpG-CAAT	28	CGCC-N_8-TATA	CpG-TATA	38
GCCAATCG	CAAT	61	TATA--CGGC	CpG-TATA	46	TATA----GGGC	CpG-TATA	25	CAAT-N_5-GCCG	CpG-CAAT	37
CAGCCAAT	CAAT	58	TATA--AGGG	CpG-TATA	44	TATA----GCGC	CpG-TATA	24	ACGT-N_8-CGCG	CpG-CRE	36
CCCCGCCC	SP1	54	TGAC--CACA	CRE	36	TATA----CCGG	CpG-TATA	21	CGCG-N_6-TGAC	CpG-CRE	35
GCCATCTT		53	TATA--CGCG	CpG-TATA	35	TATA----GCCG	CpG-TATA	19	CCCT-N_{28}-TCCC		33
TATAAAAG	TATA	53	TGAC--CACG	CRE	33	AGGC----CCCC		19	TATA-N_6-GCGG	CpG-TATA	33
GGCCAATC	CAAT	47	CAAT--GCGC	CpG-CAAT	28	CGGA----ACGT	**ETS-CRE**	18	TGAC-N_{11}-GCGC	CpG-CRE	32
CCCGGAAG	ETS	47	GGAA--GGAA	**ETS-ETS**	26	CAAT----AGCC	CpG-CAAT	18	TTAT-N_{18}-CGCC	CpG-TATA	29
ACCAATCA	CAAT	46	GGGA--TGTA		21	GCGC----ACGT	CpG-CRE	17	CAAT-N_{24}-CCGC	CpG-CAAT	28
GGAAGTGA	**ETS-CRE**	46	CCCC--CCCT		20	CCAA----CGCC	CpG-CAAT	15	CGCG-N_5-TATA	CpG-TATA	28
GCCCCGCC	SP1	44	GCGG--CAAT	CpG-CAAT	19	TGCG----CGCG		15	GGCG-N_8-TGAC	CpG-CRE	27
GCCAATCA	CAAT	44	ACCG--AGTG	ETS	18	CAAT----GCCC	CpG-CAAT	15	GGCG-N_{19}-CAAT	CpG-CAAT	26
GCGGAAGT	ETS	43	AGCG--AGTG	ETS	18	CAAT----GGCG	CpG-CAAT	14	TTAT-N_{15}-CCCG	CpG-TATA	25
CCAATGGG	CAAT	41	CGCC--AAGT	ETS	18	AGCA----AATG		14	TATA-N_6-GCCG	CpG-TATA	24
CGGCCAAT	CAAT	40	TATA--CCCG	CpG-TATA	18	TATA----GGGA	CpG-TATA	14	TATA-N_{11}-CGGC	CpG-TATA	24
CCAATCAG	CAAT	39	TATA--AGCG	CpG-TATA	17	CCGC----ATAA	CpG-TATA	13	ACCA-N_{24}-ATGG		23
AAGTGACG	CRE	37	CCCC--CCAC		17	TATA----GGCC	CpG-TATA	13	CGCC-N_7-TTAT	CpG-TATA	23
CCATCTTG		37	CCGC--TTAT	CpG-TATA	17	CAAT----GGGC	CpG-CAAT	13	TCAT-N_7-CGCC	CpG-CRE	23

Table S2 Occurrence of different length of ETS⇔CRE motifs in the human genome, promoters, proximal promoters, CpG Islands and housekeeping DNAse hypersensitive sites.

Motifs	N-mers	DNA sequence	Whole Genome	Promoter	Proximal Promoter	CpG Islands	House-keeping DHS	All DHS	Tissue-specific DHS
			# Unmasked (100%)	(-1000…500) (0.8%)	(-200…60) (0.1%)	(0.7%)	(0.2%)	(8.9%)	(8.7%)
ETS	8-mer	CGGAAGTG	16,846	1,631 (10%)	980 (6%)	1,761 (10%)	1,073 (6%)	5,068 (30%)	3,997 (24%)
ETS	9-mer	CGGAAGTGA	4,675	465 (10%)	298 (6%)	446 (10%)	343 (7%)	1,456 (31%)	1,113 (24%)
ETS	10-mer	CGGAAGTGAC	1,030	227 (22%)	162 (16%)	227 (22%)	180 (17%)	458 (44%)	278 (27%)
ETS⇔CRE	11-mer	CGGAAGTGACG	226	157 (69%)	124 (55%)	164 (73%)	134 (59%)	186 (82%)	52 (23%)
ETS⇔CRE	12-mer	CGGAAGTGACGT	93	70 (75%)	53 (57%)	71 (76%)	60 (65%)	84 (90%)	24 (26%)
ETS⇔CRE	13-mer	CGGAAGTGACGTC	33	23 (70%)	17 (52%)	25 (76%)	19 (58%)	29 (88%)	10 (30%)
ETS⇔CRE	14-mer	CGGAAGTGACGTCA	18	13 (72%)	11 (61%)	15 (83%)	12 (67%)	18 (100%)	6 (33%)
ETS⇔CRE	15-mer	CGGAAGTGACGTCAC	7	5 (71%)	4 (57%)	6 (86%)	4 (57%)	7 (100%)	3 (43%)
ETS⇔CRE	8-mer	AAGTGACG	17,396	478 (3%)	234 (1%)	451 (3%)	279 (2%)	2,647 (15%)	2,369 (14%)
ETS⇔CRE	9-mer	GAAGTGACG	4,183	289 (7%)	191 (5%)	275 (7%)	212 (5%)	1,009 (24%)	798 (19%)
ETS⇔CRE	10-mer	GGAAGTGACG	1,618	236 (15%)	176 (11%)	236 (15%)	190 (12%)	608 (38%)	418 (26%)
ETS⇔CRE	11-mer	CGGAAGTGACG	226	157 (69%)	124 (55%)	164 (73%)	134 (59%)	186 (82%)	52 (23%)
ETS⇔CRE	12-mer	CCGGAAGTGACG	100	79 (79%)	59 (59%)	83 (83%)	62 (62%)	93 (93%)	31 (31%)
ETS⇔CRE	13-mer	GCCGGAAGTGACG	33	25 (76%)	18 (55%)	28 (85%)	19 (58%)	30 (91%)	11 (33%)
ETS⇔CRE	16-mer	GCGGAAGTGACGTCAC	2	2 (100%)	2 (100%)	2 (100%)	2 (100%)	2 (100%)	0 (0%)

Table S3 Enriched GO terms (P<0.05) for the human genes that have one of the 4 ETS⇔CRE 12-mer or 13-mers ($^{C}/_{G}$CGGAAGTGACG$^{T}/_{C}$) in promoters. There are no enriched GO terms with P-value <0.05 for the genes with $^{C}/_{G}$CGGAA**GTG**ACGC in their promoters.

Motif	Sequence	GO Term	Description	Count	Background count	P-Value
ETS⇔CRE	12-mer: CGGAAGTGACGC	GO:0006281	DNA repair	5	284	8.8E-03
	12-mer: CGGAAGTGACGT	GO:0016567	protein ubiquitination	5	119	1.3E-03
		GO:0032446	protein modification by small protein conjugation	5	132	1.9E-03
		GO:0070647	protein modification by small protein conjugation or removal	5	160	3.8E-03
		GO:0044265	cellular macromolecule catabolic process	9	725	7.6E-03
		GO:0009057	macromolecule catabolic process	9	781	1.2E-02
		GO:0006396	RNA processing	9	547	1.4E-03
		GO:0006397	mRNA processing	6	321	8.9E-03
	13-mer: GCGGAAGTGACGT	GO:0051276	chromosome organization	5	485	6.0E-03
		GO:0045449	regulation of transcription	13	2601	1.3E-04
	13-mer: CCGGAAGTGACGT	GO:0016567	protein ubiquitination	3	119	1.4E-02

Table S4 Occurrence of unmethylated versions of the ETS⇔CRE motifs in the mouse genome with 24,273 promoters and proximal promoters and 16,026 CpG Islands. Unmethylated occurrences are presented in parenthesis.

Motifs	N-mers	DNA sequence	Whole Genome (# Unmethylated) (100%)	Promoter (#Unmethylated) (-1000…500) (1.18%)	Proximal Promoter (#Unmethylated) (-200…60) (0.19%)	CpG Islands (#Unmethylated) (0.34%)
ETS	8-mer	CCGGAAGT	16346 (2350)	1261 (990)	704 (643)	1362 (1286)
CRE	8-mer	TGACGTCA	14297 (1561)	578 (432)	315 (268)	599 (569)
ETS⇔CRE	12-mer	CGGAAGTGACGT	89 (67)	37 (36)	31 (31)	60 (60)
ETS⇔CRE	12-mer	CGGAAGTGACGC	82 (68)	35 (35)	33 (33)	68 (67)
ETS⇔CRE	13-mer	GCGGAAGTGACGT	21 (21)	10 (10)	7 (7)	19 (19)
ETS⇔CRE	13-mer	GCGGAAGTGACGC	28 (25)	15 (15)	13 (13)	26 (25)
ETS⇔CRE	13-mer	CCGGAAGTGACGT	45 (34)	21 (20)	18 (18)	29 (29)
ETS⇔CRE	13-mer	CCGGAAGTGACGC	42 (36)	22 (22)	17 (17)	35 (35)
ETS⇔CRE	15-mer	CCGGAAGTGACGTCA	12 (8)	8 (7)	7 (7)	7 (7)
N-CG-N_7-CG	12-mer	ACGCACACACCG	42 (8)	3 (3)	5 (4)	0 (0)
N_2-CG-N_7-CG	13-mer	CACGCACACACCG	32 (4)	2 (2)	3 (2)	0 (0)
