# Supplementary material for: Overlapping ETS and CRE Motifs (G/CCGGAAGTGACGTCA) Preferentially Bound by GABPα and CREB Proteins
Source: G3 (Bethesda). 2012 Oct 1;2(10):1243–56. doi: 10.1534/g3.112.004002 (PMC3464117; doi:10.1534/g3.112.004002)
Supplement: Supporting Information [file supp_2.10.1243_TableS4.pdf]

Table S4 Occurrence of unmethylated versions of the ETS⇔CRE motifs in the mouse genome with 24,273 promoters and proximal promoters and 16,026 CpG Islands. Unmethylated occurrences are presented in parenthesis.

Motifs	N-mers	DNA sequence	Whole Genome (# Unmethylated) (100%)	Promoter (#Unmethylated) (-1000...500) (1.18%)	Proximal Promoter (#Unmethylated) (-200...60) (0.19%)	CpG Islands (#Unmethylated) (0.34%)
ETS	8-mer	CCGGAAGT	16346 (2350)	1261 (990)	704 (643)	1362 (1286)
CRE	8-mer	TGACGTCA	14297 (1561)	578 (432)	315 (268)	599 (569)
ETS⇔CRE	12-mer	CGGAAGTGACGT	89 (67)	37 (36)	31 (31)	60 (60)
ETS⇔CRE	12-mer	CGGAAGTGACGC	82 (68)	35 (35)	33 (33)	68 (67)
ETS⇔CRE	13-mer	GCGGAAGTGACGT	21 (21)	10 (10)	7 (7)	19 (19)
ETS⇔CRE	13-mer	GCGGAAGTGACGC	28 (25)	15 (15)	13 (13)	26 (25)
ETS⇔CRE	13-mer	CCGGAAGTGACGT	45 (34)	21 (20)	18 (18)	29 (29)
ETS⇔CRE	13-mer	CCGGAAGTGACGC	42 (36)	22 (22)	17 (17)	35 (35)
ETS⇔CRE	15-mer	CCGGAAGTGACGTCA	12 (8)	8 (7)	7 (7)	7 (7)
N-CG-N_7-CG	12-mer	ACGCACACACCG	42 (8)	3 (3)	5 (4)	0 (0)
N_2-CG-N_7-CG	13-mer	CACGCACACACCG	32 (4)	2 (2)	3 (2)	0 (0)
