# Supplementary material for: Overlapping ETS and CRE Motifs (G/CCGGAAGTGACGTCA) Preferentially Bound by GABPα and CREB Proteins
Source: G3 (Bethesda). 2012 Oct 1;2(10):1243–56. doi: 10.1534/g3.112.004002 (PMC3464117; doi:10.1534/g3.112.004002)
Supplement: Supporting Information [file supp_2.10.1243_FigureS1.pdf]

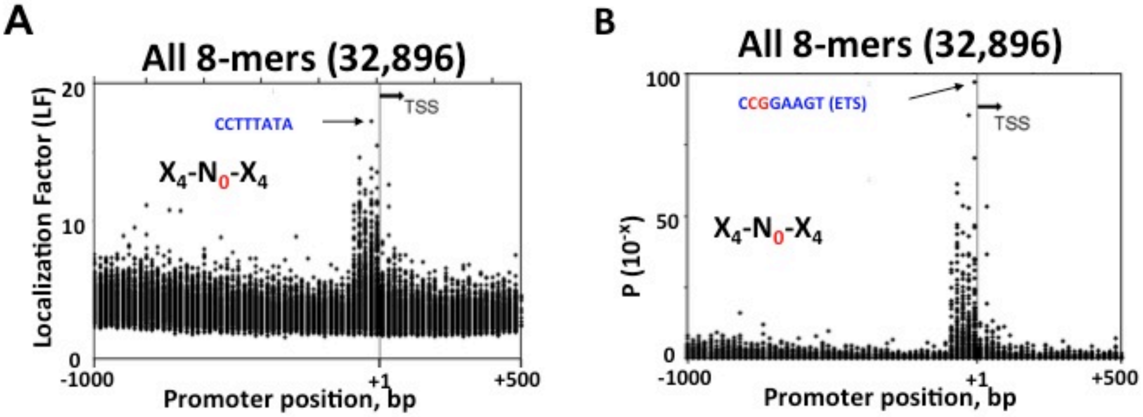

Figure S1 Localization of 8-mers in human promoters. **A)** Localization Factor (LF), a measure of non-random distribution of a DNA sequence, for 32,896 continuous 8-mers (X_4-N_0-X_4). For each 8-mer, the distribution in 17,143 human promoters (-1,000 bp to +500 bp) aligned relative to the TSS was determined and plotted in the most abundant 20 bp bin. Some sequences are preferentially localized near the TSS. **B)** Probability ($p=10^{-x}$) that an 8-mer have a non-random distribution is plotted in the most abundant bin for all 8-mers.
