# Supplementary material for: Overlapping ETS and CRE Motifs (G/CCGGAAGTGACGTCA) Preferentially Bound by GABPα and CREB Proteins
Source: G3 (Bethesda). 2012 Oct 1;2(10):1243–56. doi: 10.1534/g3.112.004002 (PMC3464117; doi:10.1534/g3.112.004002)
Supplement: Supporting Information [file supp_2.10.1243_FigureS2.pdf]

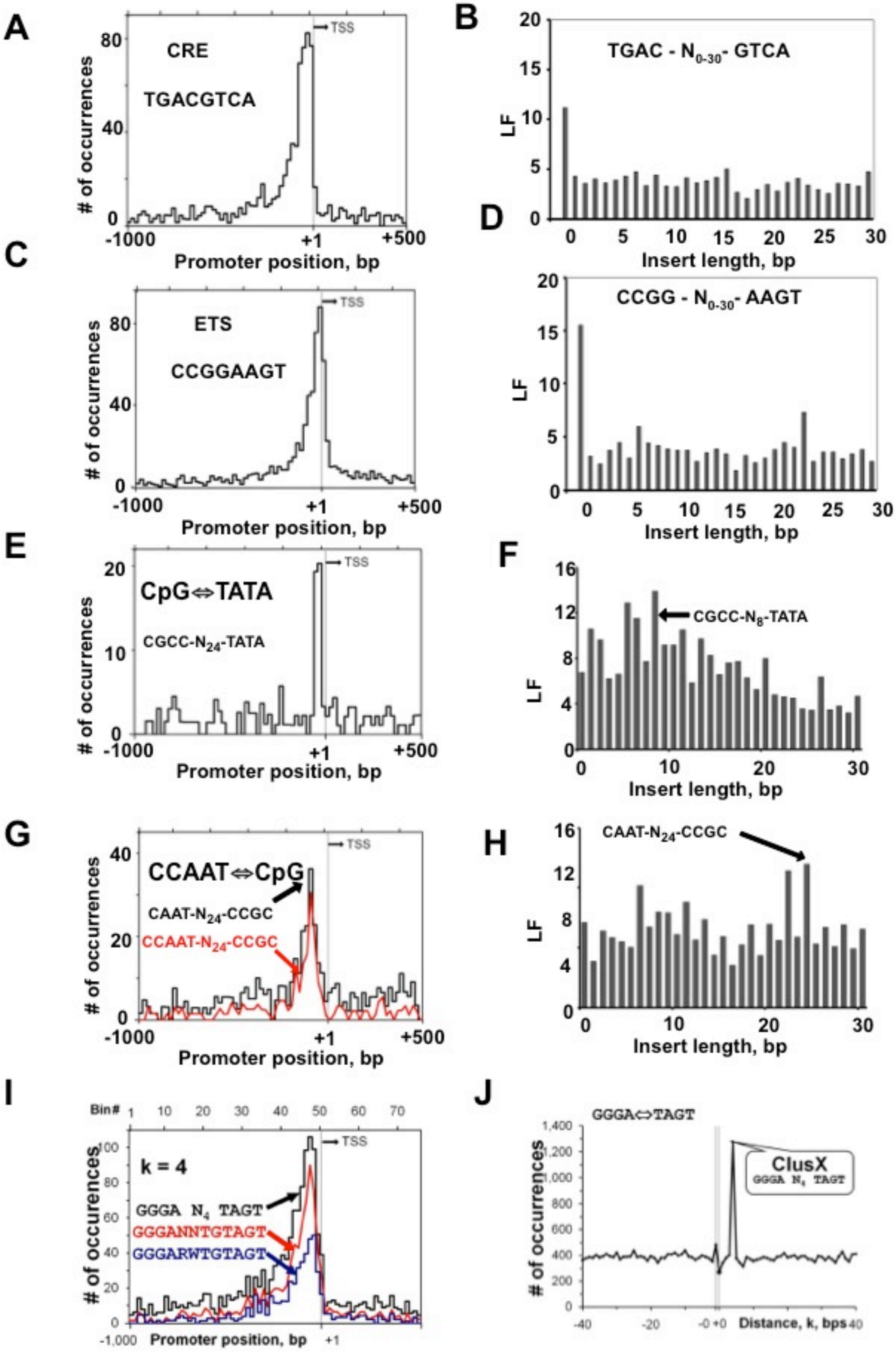

Figure S2 **A)** Distribution of the CRE 8-mer TGACGTCA in human promoters. **B)** LF for CRE 8-mer with insert length ranging from 0-bps to 30-bps (TGAC-$N_{0\text{-}30}$-GTCA). **C)** Distribution of the ETS TFBS (CCGGAAGT) in promoters counting occurrence in 20 bp bins. **D)** LF for ETS 8-mer (CCGG-$N_{0\text{-}30}$-AAGT) with insert length ranging from 0-bps to 30-bps. The 8-mer CCGGAAGT preferentially localize in promoters when the two 4-mers are abutted. High LF values are also observed at additional insert

length, including CCGG-N_6-AAGT, which represents the ETS⇔ETS motif and CCGG-N_{23}-AAGT. **E)** Distribution of the split 8-mer CGCC-N_{24}-TATA in promoters. **F)** LF for CGCC-$N_{0\text{-}30}$-TATA with insert length ranging from 0-bps to 30-bps. **G)** Distribution of the split 8-mer CAAT-N_{24}-CCGC in human promoters. The most localizing motif is the split 9-mer CCAAT-N_{24}-CCGC marked in red. **H)** LF for CAAT-$N_{0\text{-}30}$-CCGC with insert length from 0-bps to 30-bps. **I)** Distribution of the split 8-mer GGGA-N_4-TAGT and more localizing split 10-mer GGGA-N_2-TGTAGT. **J)** Occurrences of split 8-mer GGGA-$N_{0\text{-}40}$-TAGT with insert length ranging from 0-bps to 40-bps. The reverse order of the two 4-mers TAGT-$N_{0\text{-}40}$-GGGA is shown with negative values.
