# Supplementary material for: Overlapping ETS and CRE Motifs (G/CCGGAAGTGACGTCA) Preferentially Bound by GABPα and CREB Proteins
Source: G3 (Bethesda). 2012 Oct 1;2(10):1243–56. doi: 10.1534/g3.112.004002 (PMC3464117; doi:10.1534/g3.112.004002)
Supplement: Supporting Information [file supp_2.10.1243_FigureS3.pdf]

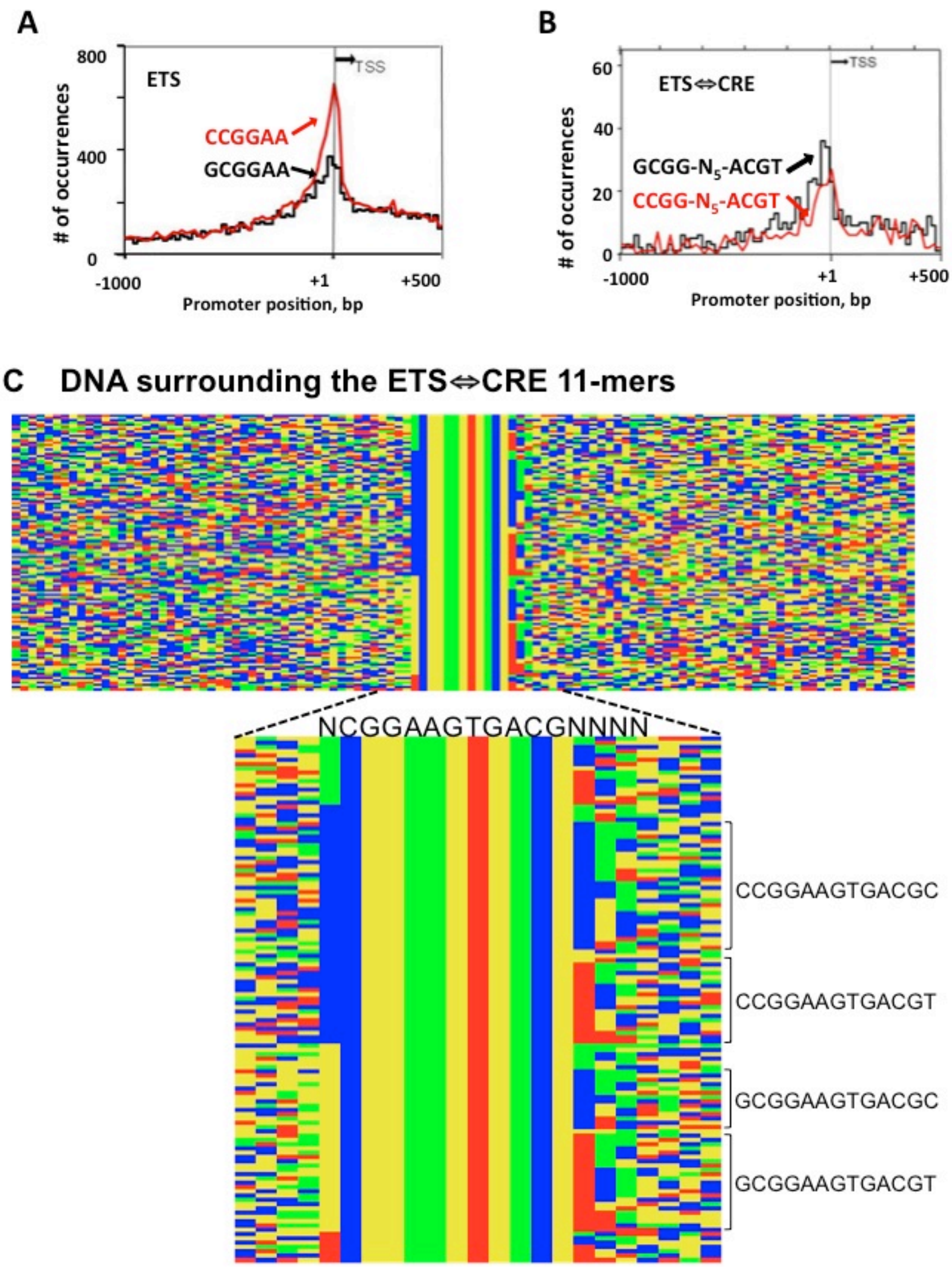

Figure S3 **A)** Distribution of the two ETS TFBS (CCGGAA and GCGGAA). **B)** Distribution of the two split 8-mers GCGG-N_5-ACGT and CCGG-N_5-ACGT representing the ETS⇔CRE motif. **C**) Color representation of the sequences surrounding the 134 ETS⇔CRE 11-mers that occur in housekeeping DHSs with C=blue, G=yellow, A=green and T=red. The core part of the ETS⇔CRE motif is shown in inset. Sequences were grouped based on the nucleotides as shown by numbers in bold: **1**CGGAAGTGACG**2345** where the numbers represent the order of grouping.
