# Supplementary material for: Overlapping ETS and CRE Motifs (G/CCGGAAGTGACGTCA) Preferentially Bound by GABPα and CREB Proteins
Source: G3 (Bethesda). 2012 Oct 1;2(10):1243–56. doi: 10.1534/g3.112.004002 (PMC3464117; doi:10.1534/g3.112.004002)
Supplement: Supporting Information [file supp_2.10.1243_FigureS4.pdf]

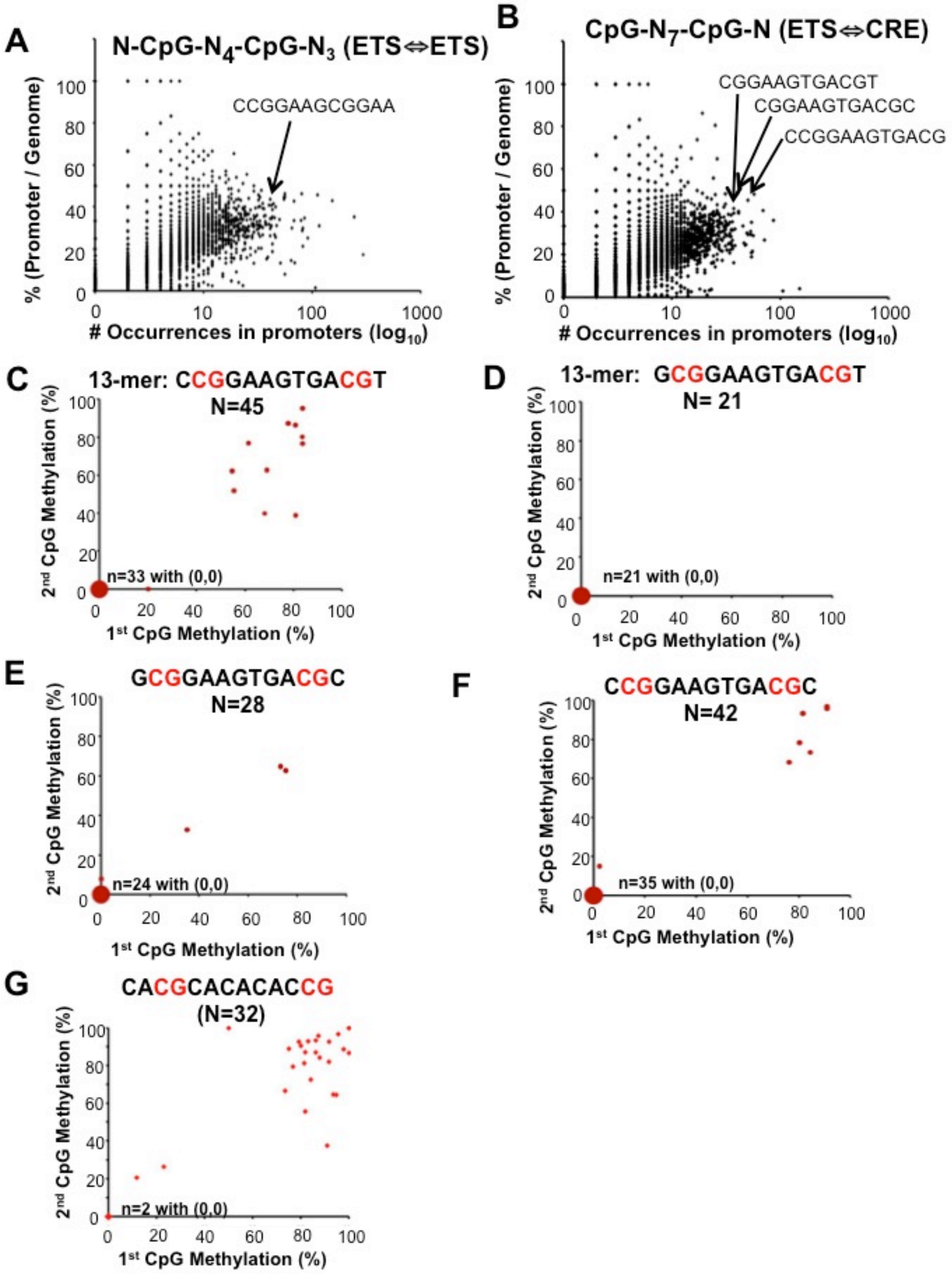

Figure S4 **A)** Occurrence in mouse promoters compared to the genome of all split 8-mer containing two CGs separated by 4-bps (N-CG-N_4-CG-N_3) as is observed in the ETS⇔ETS motif which is labeled. **B)** Occurrence in mouse promoters compared to the genome of all split 8-mer containing two CGs separated by 7-bps CG-N_7-CG-N) as is observed in the ETS⇔CRE motif which is labeled **C-F)** Methylation status in mouse dermal fibroblasts of the 4 ETS⇔CRE 13-mers $^{C}/_{G}$CGGAA**GTG**ACG$^{T}/_{C}$. Percent methylations of 1st and 2nd CpGs are plotted for each 4 motifs. The majority of occurrences have no CpG methylation on either CpG. **G)** Methylation of 1st and 2nd CGs for the 13-mer CACGCACACACCG with pairs of CpG separated by 7-bps showing both the CpGs in the motif are mostly methylated in dermal fibroblasts.
