# Supplementary material for: Overlapping ETS and CRE Motifs (G/CCGGAAGTGACGTCA) Preferentially Bound by GABPα and CREB Proteins
Source: G3 (Bethesda). 2012 Oct 1;2(10):1243–56. doi: 10.1534/g3.112.004002 (PMC3464117; doi:10.1534/g3.112.004002)
Supplement: Supporting Information [file supp_2.10.1243_FigureS5.pdf]

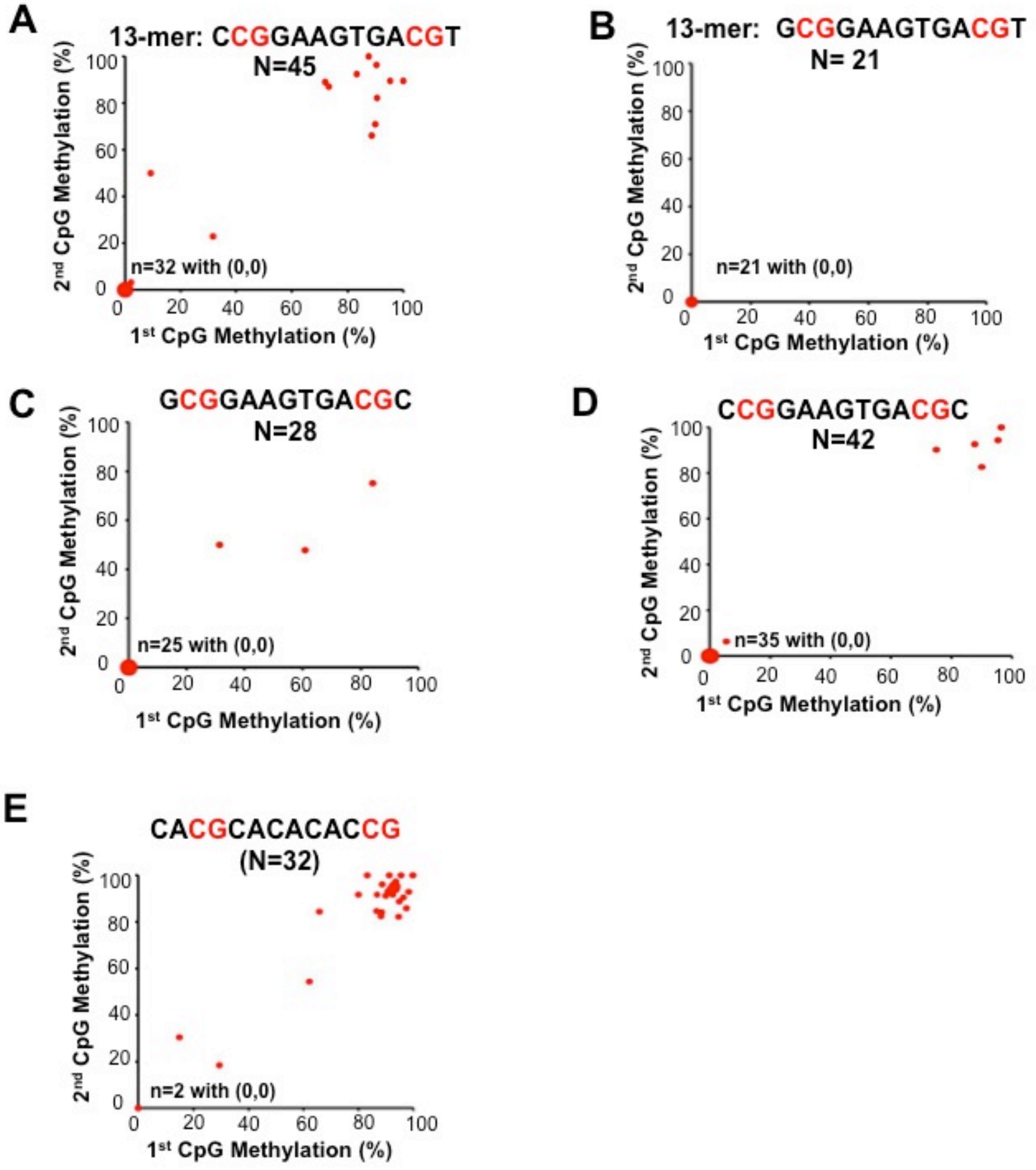

Figure S5 A-D) Methylation status in mouse primary keratinocytes of the 4 ETS⇔CRE 13-mers $^{C}/_{G}$CGGAA**GTG**ACG$^{T}/_{C}$. Percent methylations of 1st and 2nd CpGs are plotted. The majority of occurrences have no CpG methylation on either CpG. **E)** Methylation of 1st and 2nd CGs for the 13-mer CACGCACACACCG with pairs of CpG separated by 7-bps showing both the CpGs in the motif are mostly methylated in keratinocytes.
