# Supplementary material for: Overlapping ETS and CRE Motifs (G/CCGGAAGTGACGTCA) Preferentially Bound by GABPα and CREB Proteins
Source: G3 (Bethesda). 2012 Oct 1;2(10):1243–56. doi: 10.1534/g3.112.004002 (PMC3464117; doi:10.1534/g3.112.004002)
Supplement: Supporting Information [file supp_2.10.1243_FigureS6.pdf]

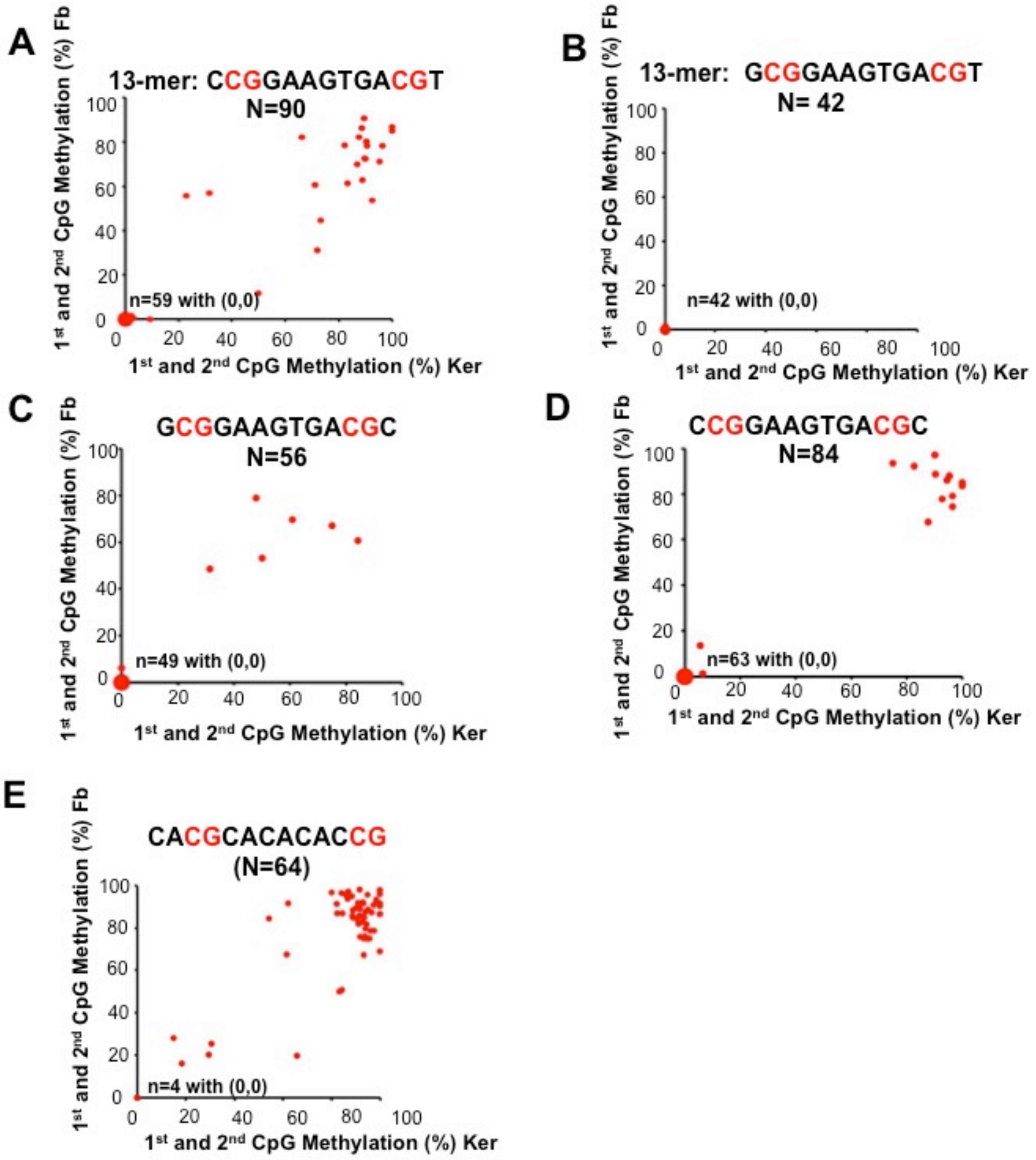

Figure S6 A-D) Comparison in methylation status of both the CpGs in 4 ETS⇔CRE 13-mers $^{C}/_{G}$CGGAA**GTG**ACG$^{T}/_{C}$ in mouse dermal fibroblasts and keratinocytes. **E)** Methylation of 1st and 2nd CGs for the 13-mer CACGCACACACCG in primary dermal fibroblasts and keratinocytes.
