# Supplementary material for: Overlapping ETS and CRE Motifs (G/CCGGAAGTGACGTCA) Preferentially Bound by GABPα and CREB Proteins
Source: G3 (Bethesda). 2012 Oct 1;2(10):1243–56. doi: 10.1534/g3.112.004002 (PMC3464117; doi:10.1534/g3.112.004002)
Supplement: Supporting Information [file supp_2.10.1243_TableS1.pdf]

**Table S1 The Localization Factor (LF) for all continuous ($X_4$-$N_0$-$X_4$) and split 8-mers ($X_4$-$N_{1\text{-}30}$-$X_4$) was determined.** Next, the probability of a non-random distribution was determined and the top 20 for different insert length was determined. For column $X_4$-$N_{5\text{-}30}$-$X_4$, the insert length that produced the highest LF is presented. The most localized split 8-mers are further extended to determine the TFBS or overlapping TFBS and are presented in the column next to the sequence motifs. Two sequences representing the ETS⇔ETS motif and the ETS⇔CRE motif are highlighted in red.

| $X_4$-$N_0$-$X_4$ | | | $X_4$-$N_2$-$X_4$ | | | $X_4$-$N_4$-$X_4$ | | | $X_4$-$N_{5\text{-}30}$-$X_4$ | | |
|---|---|---|---|---|---|---|---|---|---|---|---|
| Sequence | Predicted TFBS | P-value | Sequence | Predicted TFBS | P-value | Sequence | Predicted TFBS | P-value | Sequence | Predicted TFBS | P-value |
| CCGGAAGT | ETS | 97 | GTGA--TCAC | CRE | 56 | TATA----CGGC | CpG-TATA | 92 | ACGT- $N_9$-GCGC | CpG-CRE | 56 |
| TATAAAGG | TATA | 85 | CGGA--TGAC | **ETS-CRE** | 47 | TATA----CGGG | CpG-TATA | 36 | GCGC-$N_7$-TGAC | CpG-CRE | 44 |
| CGGAAGTG | ETS | 70 | GCCC--CCCC | SP1 | 47 | CAAT----GCGC | CpG-CAAT | 28 | CGCC-$N_8$-TATA | CpG-TATA | 38 |
| GCCAATCG | CAAT | 61 | TATA--CGGC | CpG-TATA | 46 | TATA----GGGC | CpG-TATA | 25 | CAAT-$N_5$-GCCG | CpG-CAAT | 37 |
| CAGCCAAT | CAAT | 58 | TATA--AGGG | CpG-TATA | 44 | TATA----GCGC | CpG-TATA | 24 | ACGT-$N_8$-CGCG | CpG-CRE | 36 |
| CCCCGCCC | SP1 | 54 | TGAC--CACA | CRE | 36 | TATA----CCGG | CpG-TATA | 21 | CGCG-$N_6$-TGAC | CpG-CRE | 35 |
| GCCATCTT | | 53 | TATA--CGCG | CpG-TATA | 35 | TATA----GCCG | CpG-TATA | 19 | CCCT-$N_{28}$-TCCC | | 33 |
| TATAAAAG | TATA | 53 | TGAC--CACG | CRE | 33 | AGGC----CCCC | | 19 | TATA-$N_6$-GCGG | CpG-TATA | 33 |
| GGCCAATC | CAAT | 47 | CAAT--GCGC | CpG-CAAT | 28 | CGGA----ACGT | **ETS-CRE** | 18 | TGAC-$N_{11}$-GCGC | CpG-CRE | 32 |
| CCCGGAAG | ETS | 47 | GGAA--GGAA | **ETS-ETS** | 26 | CAAT----AGCC | CpG-CAAT | 18 | TTAT-$N_{18}$-CGCC | CpG-TATA | 29 |
| ACCAATCA | CAAT | 46 | GGGA--TGTA | | 21 | GCGC----ACGT | CpG-CRE | 17 | CAAT-$N_{24}$-CCGC | CpG-CAAT | 28 |
| GGAAGTGA | **ETS-CRE** | 46 | CCCC--CCCT | | 20 | CCAA----CGCC | CpG-CAAT | 15 | CGCG-$N_5$-TATA | CpG-TATA | 28 |
| GCCCCGCC | SP1 | 44 | GCGG--CAAT | CpG-CAAT | 19 | TGCG----CGCG | | 15 | GGCG-$N_8$-TGAC | CpG-CRE | 27 |
| GCCAATCA | CAAT | 44 | ACCG--AGTG | ETS | 18 | CAAT----GCCC | CpG-CAAT | 15 | GGCG-$N_{19}$-CAAT | CpG-CAAT | 26 |
| GCGGAAGT | ETS | 43 | AGCG--AGTG | ETS | 18 | CAAT----GGCG | CpG-CAAT | 14 | TTAT-$N_{15}$-CCCG | CpG-TATA | 25 |
| CCAATGGG | CAAT | 41 | CGCC--AAGT | ETS | 18 | AGCA----AATG | | 14 | TATA-$N_6$-GCCG | CpG-TATA | 24 |
| CGGCCAAT | CAAT | 40 | TATA--CCCG | CpG-TATA | 18 | TATA----GGGA | CpG-TATA | 14 | TATA-$N_{11}$-CGGC | CpG-TATA | 24 |
| CCAATCAG | CAAT | 39 | TATA--AGCG | CpG-TATA | 17 | CCGC----ATAA | CpG-TATA | 13 | ACCA-$N_{24}$-ATGG | | 23 |
| AAGTGACG | CRE | 37 | CCCC--CCAC | | 17 | TATA----GGCC | CpG-TATA | 13 | CGCC-$N_7$-TTAT | CpG-TATA | 23 |
| CCATCTTG | | 37 | CCGC--TTAT | CpG-TATA | 17 | CAAT----GGGC | CpG-CAAT | 13 | TCAT-$N_7$-CGCC | CpG-CRE | 23 |
