# Supplementary material for: Overlapping ETS and CRE Motifs (G/CCGGAAGTGACGTCA) Preferentially Bound by GABPα and CREB Proteins
Source: G3 (Bethesda). 2012 Oct 1;2(10):1243–56. doi: 10.1534/g3.112.004002 (PMC3464117; doi:10.1534/g3.112.004002)
Supplement: Supporting Information [file supp_2.10.1243_TableS2.pdf]

Table S2 Occurrence of different length of ETS⇔CRE motifs in the human genome, promoters, proximal promoters, CpG Islands and housekeeping DNAse hypersensitive sites.

Motifs	N-mers	DNA sequence	Whole Genome	Promoter	Proximal Promoter	CpG Islands	House-keeping DHS	All DHS	Tissue-specific DHS
			# Unmasked (100%)	(-1000…500) (0.8%)	(-200…60) (0.1%)	(0.7%)	(0.2%)	(8.9%)	(8.7%)
ETS	8-mer	CGGAAGTG	16,846	1,631 (10%)	980 (6%)	1,761 (10%)	1,073 (6%)	5,068 (30%)	3,997 (24%)
ETS	9-mer	CGGAAGTGA	4,675	465 (10%)	298 (6%)	446 (10%)	343 (7%)	1,456 (31%)	1,113 (24%)
ETS	10-mer	CGGAAGTGAC	1,030	227 (22%)	162 (16%)	227 (22%)	180 (17%)	458 (44%)	278 (27%)
ETS⇔CRE	11-mer	CGGAAGTGACG	226	157 (69%)	124 (55%)	164 (73%)	134 (59%)	186 (82%)	52 (23%)
ETS⇔CRE	12-mer	CGGAAGTGACGT	93	70 (75%)	53 (57%)	71 (76%)	60 (65%)	84 (90%)	24 (26%)
ETS⇔CRE	13-mer	CGGAAGTGACGTC	33	23 (70%)	17 (52%)	25 (76%)	19 (58%)	29 (88%)	10 (30%)
ETS⇔CRE	14-mer	CGGAAGTGACGTCA	18	13 (72%)	11 (61%)	15 (83%)	12 (67%)	18 (100%)	6 (33%)
ETS⇔CRE	15-mer	CGGAAGTGACGTCAC	7	5 (71%)	4 (57%)	6 (86%)	4 (57%)	7 (100%)	3 (43%)
ETS⇔CRE	8-mer	AAGTGACG	17,396	478 (3%)	234 (1%)	451 (3%)	279 (2%)	2,647 (15%)	2,369 (14%)
ETS⇔CRE	9-mer	GAAGTGACG	4,183	289 (7%)	191 (5%)	275 (7%)	212 (5%)	1,009 (24%)	798 (19%)
ETS⇔CRE	10-mer	GGAAGTGACG	1,618	236 (15%)	176 (11%)	236 (15%)	190 (12%)	608 (38%)	418 (26%)
ETS⇔CRE	11-mer	CGGAAGTGACG	226	157 (69%)	124 (55%)	164 (73%)	134 (59%)	186 (82%)	52 (23%)
ETS⇔CRE	12-mer	CCGGAAGTGACG	100	79 (79%)	59 (59%)	83 (83%)	62 (62%)	93 (93%)	31 (31%)
ETS⇔CRE	13-mer	GCCGGAAGTGACG	33	25 (76%)	18 (55%)	28 (85%)	19 (58%)	30 (91%)	11 (33%)
ETS⇔CRE	16-mer	GCGGAAGTGACGTCAC	2	2 (100%)	2 (100%)	2 (100%)	2 (100%)	2 (100%)	0 (0%)
