# Supplementary material for: Overlapping ETS and CRE Motifs (G/CCGGAAGTGACGTCA) Preferentially Bound by GABPα and CREB Proteins
Source: G3 (Bethesda). 2012 Oct 1;2(10):1243–56. doi: 10.1534/g3.112.004002 (PMC3464117; doi:10.1534/g3.112.004002)
Supplement: Supporting Information [file supp_2.10.1243_TableS3.pdf]

Table S3 Enriched GO terms (P<0.05) for the human genes that have one of the 4 ETS⇔CRE 12-mer or 13-mers ($^{C}/_{G}$CGGAAGTGACG$^{T}/_{C}$) in promoters. There are no enriched GO terms with P-value <0.05 for the genes with $^{C}/_{G}$CGGAA**GTG**ACGC in their promoters.

Motif	Sequence	GO Term	Description	Count	Background count	P-Value
ETS⇔CRE	**12-mer: CGGAAGTGACGC**	GO:0006281	DNA repair	5	284	8.8E-03
	**12-mer: CGGAAGTGACGT**	GO:0016567	protein ubiquitination	5	119	1.3E-03
		GO:0032446	protein modification by small protein conjugation	5	132	1.9E-03
		GO:0070647	protein modification by small protein conjugation or removal	5	160	3.8E-03
		GO:0044265	cellular macromolecule catabolic process	9	725	7.6E-03
		GO:0009057	macromolecule catabolic process	9	781	1.2E-02
		GO:0006396	RNA processing	9	547	1.4E-03
		GO:0006397	mRNA processing	6	321	8.9E-03
	**13-mer: GCGGAAGTGACGT**	GO:0051276	chromosome organization	5	485	6.0E-03
		GO:0045449	regulation of transcription	13	2601	1.3E-04
	**13-mer: CCGGAAGTGACGT**	GO:0016567	protein ubiquitination	3	119	1.4E-02
